# Rocking-Chair Proton Batteries with Conducting Redox Polymer Active Materials and Protic Ionic Liquid Electrolytes

**DOI:** 10.1021/acsami.1c01353

**Published:** 2021-04-15

**Authors:** Huan Wang, Rikard Emanuelsson, Christoffer Karlsson, Patric Jannasch, Maria Strømme, Martin Sjödin

**Affiliations:** † Nanotechnology and Functional Materials, Department of Materials Science and Engineering, The Ångström Laboratory, Uppsala University, P.O. Box 35, SE-751 03 Uppsala, Sweden; ‡ Centre for Analysis and Synthesis, Department of Chemistry, Lund University, P.O. Box 124, SE-221 00 Lund, Sweden

**Keywords:** polymerization, conducting polymers, quinone, ionic liquid, organic battery, proton battery

## Abstract

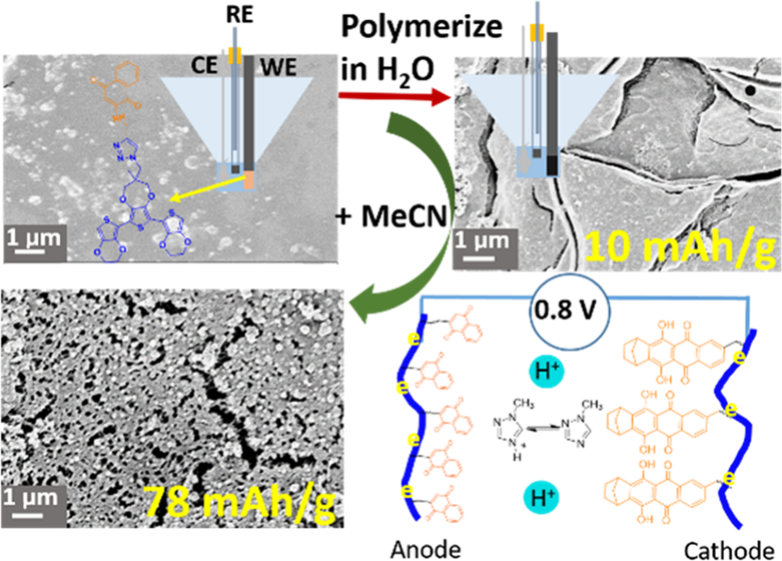

Rechargeable batteries that use redox-active organic compounds are currently considered an energy storage technology for the future. Functionalizing redox-active groups onto conducting polymers to make conducting redox polymers (CRPs) can effectively solve the low conductivity and dissolution problems of redox-active compounds. Here, we employ a solution-processable postdeposition polymerization (PDP) method, where the rearrangements ensured by partial dissolution of intermediated trimer during polymerization were found significant to produce high-performance CRPs. We show that quinizarin (Qz)- and naphthoquinone (NQ)-based CRPs can reach their theoretical capacity through optimization of the polymerization conditions. Combining the two CRPs, with the Qz-CRP as a cathode, the NQ-CRP as an anode, and a protic ionic liquid electrolyte, yields a 0.8 V proton rocking-chair battery. The conducting additive-free all-organic proton battery exhibits a capacity of 62 mAh/g and a capacity retention of 80% after 500 cycles using rapid potentiostatic charging and galvanostatic discharge at 4.5 C.

## 1 Introduction

The development of large-scale power systems such as electric vehicles and smart grids escalates the demand for energy storage technologies, such as batteries. Most batteries, however, depend on unsustainable inorganic materials and suffer from environmental issues and high CO_2_ footprints.^1,2^ Significant research focus has therefore been put on replacing inorganic energy storage materials used in traditional lithium-ion batteries with sustainable, earth-abundant, low CO_2_ footprint, and cheap organic materials (containing C, H, O, N) that, in addition, would provide simplified end-of-use treatments.^3−12^

The quinones, as some of the simplest carbonyl compounds, are particularly attractive as charge storage components due to their high specific capacities and reversible and fast two-electron (2e) redox reactions.^13−15^ Moreover, electron-withdrawing and electron-donating substituents as well as fused aromatic rings can effectively tune the quinone redox potential by altering the electron density of the quinone core.^16−21^ In addition, the versatile quinone redox chemistry is compatible with several different cycling cations, including alkali metal cations (e.g., Li^+^, Na^+^), organic ammonium cations and protons, as well as with different solvents.^22−24^

Utilizing protons as cycling ions is particularly interesting as the proton is light and abundant and has the highest diffusion coefficient known to date.^25−27^ There has been significant progress in the development of proton batteries during the last few years with respect to voltage output, discharge capacity, and stability. Yao’s group used pyrene-4,5,9,10-tetraone as an anode together with PbO_2_ as a cathode, making a 1.2 V aqueous proton battery with a capacity retention of 96% after 1500 cycles.^28^ In our previous report, naphthoquinone (NQ) and hydroquinone were functionalized onto a conducting polymer, making a conducting additive-free 0.4 V aqueous proton battery, showing 85% capacity retention after 500 cycles.^29^ Honma’s group instead combined an anthraquinone anode with a tetrachlorohydroquinone cathode in an aqueous battery that extended the voltage output to 0.6 V.^30^ Using symmetric 2,3-dimethyl-quinizarin as both a cathode and an anode, Aziz’s group presented a battery with a voltage output (1.16 V) that closely matched the stability window of water (1.23 V).^31^ The capacity retention was, however, rather limited, with 47% of the initial capacity remaining after 100 cycles. The limited stability can be traced to the water solvent as several reports show that in the oxidized state quinone can react with water through Michael addition.^31−35^ By replacing the water solvent, it should therefore be possible to increase the stability of proton batteries by suppressing the Michael addition reaction. This could be done in organic protic electrolytes^36^ or using nonstoichiometric protic ionic liquids,^37^ which also could allow a potential window over 1.23 V.^38−40^ We have previously shown that the use of nonaqueous solvents significantly extends the possibility to tune quinone redox potentials by substitution since, in aqueous solutions, specific interactions with water molecules counteract the effect of substituents.^41^ Finally, protic ionic liquids may provide a solution for redox-active materials that show poor wettability in water electrolytes. One type of protic ionic liquids is composed of positively charged, protonated nitrogen heterocycles and a suitable charge-compensating anion. In nonstoichiometric protic ionic liquids, only a portion of the nitrogen heterocycle moieties is protonated, and hence, the electrolyte can act as both a proton acceptor and a proton donor and can thus sustain proton-coupled redox reactions. The acidity and relative fraction of the protonated (acid) and the unprotonated (base) heterocycle moieties can be used to tune both the kinetics and the energetics of proton-coupled redox reactions.^37^

**Scheme 1:**
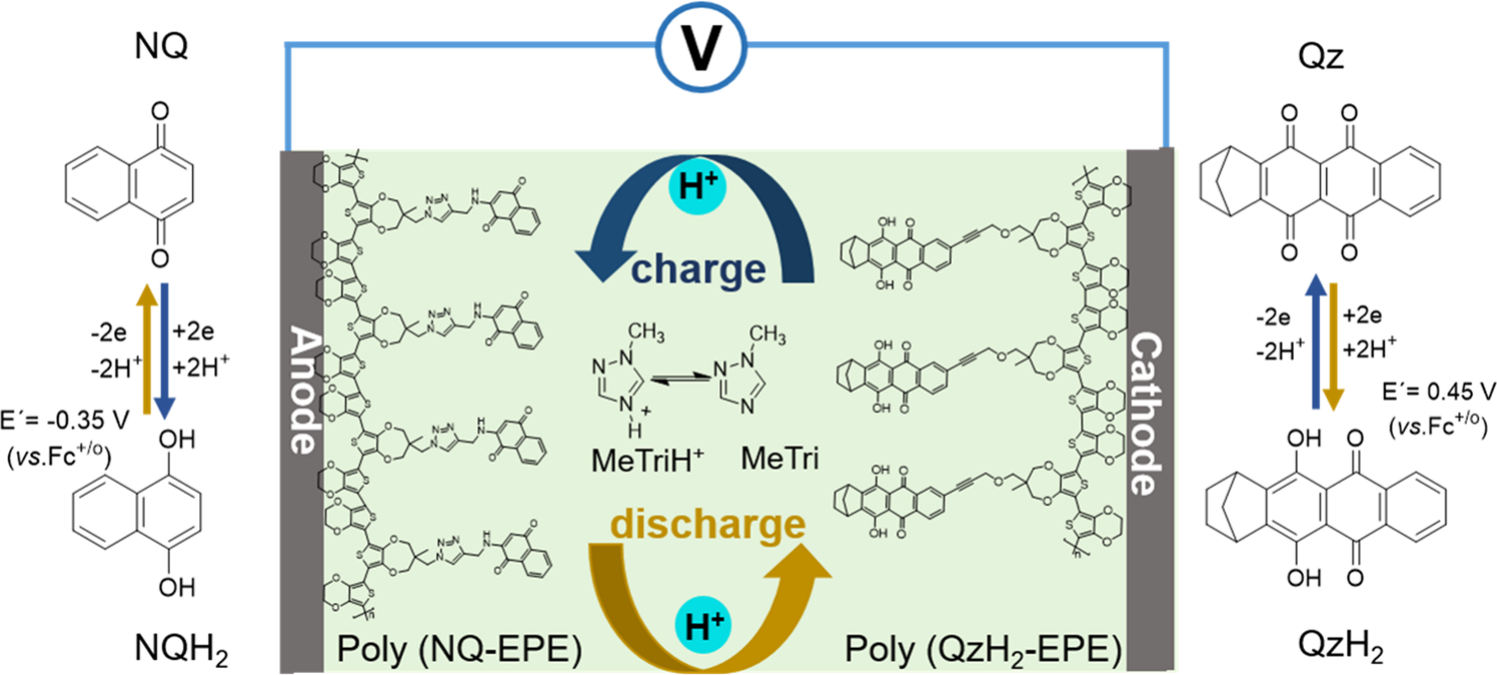
Schematic Illustration of the Working Principle of a NQ–Qz Rocking-Chair Proton Battery

Herein, a 1-methyl-1,2,4-triazole (MeTri)-based ionic liquid (p*K*_a_ = 3.2), in which the potential of capacity-carrying pendants is within the conducting region of the polymer backbone, was used as an electrolyte together with quinone-based conducting redox polymer (CRP) electrode materials. Capacity-carrying quinizarin (1,4-dihydroxyanthraquinone, Qz) and NQ pendants covalently attached onto a conducting polymer backbone were used as a cathode and an anode, respectively. The conducting polymer backbone provides electron transport pathways for the pendants’ redox reactions and also prevents the dissolution of pendants. A conducting additive-free all-organic proton rocking-chair battery was thereby fabricated (Scheme 1).

## 2 Results and Discussion

### 2.1 Postdeposition Electropolymerization

The prepared trimer film was polymerized using postdeposition polymerization (PDP),^29^ which allows the full utilization of starting materials as opposed to traditional polymerization from a monomer solution, where most starting materials remain unreacted. In this method, a layer of the repeating unit precursor is immersed in an electrolyte solution (the polymerization solution), where the layer is oxidized by oxidants in the electrolyte solution or by electrochemically applying a sufficiently positive potential to the layer.^29^ An important prerequisite for this method to work is that the initial precursor layer does not dissolve in the polymerization solution. This can be achieved by the choice of polymerization solution as well as using precursors that interact strongly with each other. Here, we use thiophene-based trimer structures with extended π-systems that are likely to interact by π–π stacking. The utilization of trimeric precursors also ensures a lower oxidation (polymerization) potential compared to monomeric analogues (above 1 V vs Fc^+/0^), allowing for milder polymerization conditions to be used. In contrast to monomeric analogues, the trimeric precursors also exhibit electronic conductivity upon oxidation, allowing for electronic communication through the trimer layer.

**Figure 1:**
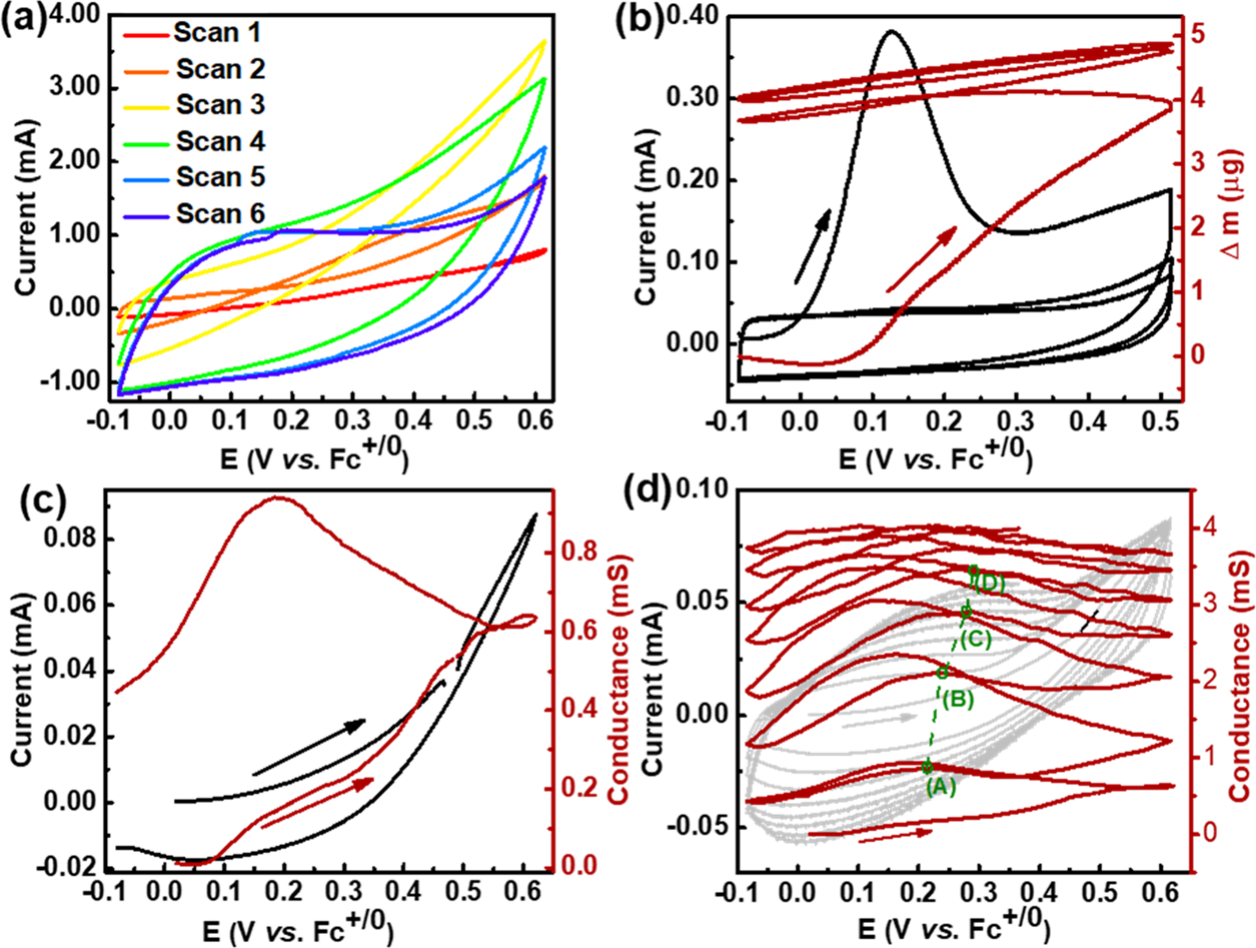
(a) Cyclic voltammograms recorded during polymerization of 0.5 mg of NQ-EPE deposited on a glassy carbon plate at 20 mV/s. (b) Cyclic voltammograms (black) and corresponding mass change (red brown) during the polymerization of 10 μg of NQ-EPE on an EQCM Au electrode at 20 mV/s. The arrows indicate the first anodic scan. Conductance (red brown) and cyclic voltammograms (black) of the (c) first polymerization scan and (d) first seven polymerization scans of 10 μg of NQ-EPE on the IDA Au electrode at 50 mV/s. Points A, B, C, and D represent the potentials where the conductance reaches its maximum value during an anodic scan. The electrolyte in the above experiments is 0.1 M MeTriHTFSI/MeCN/H_2_O (vol MeCN: 67%).

**Scheme 2:**
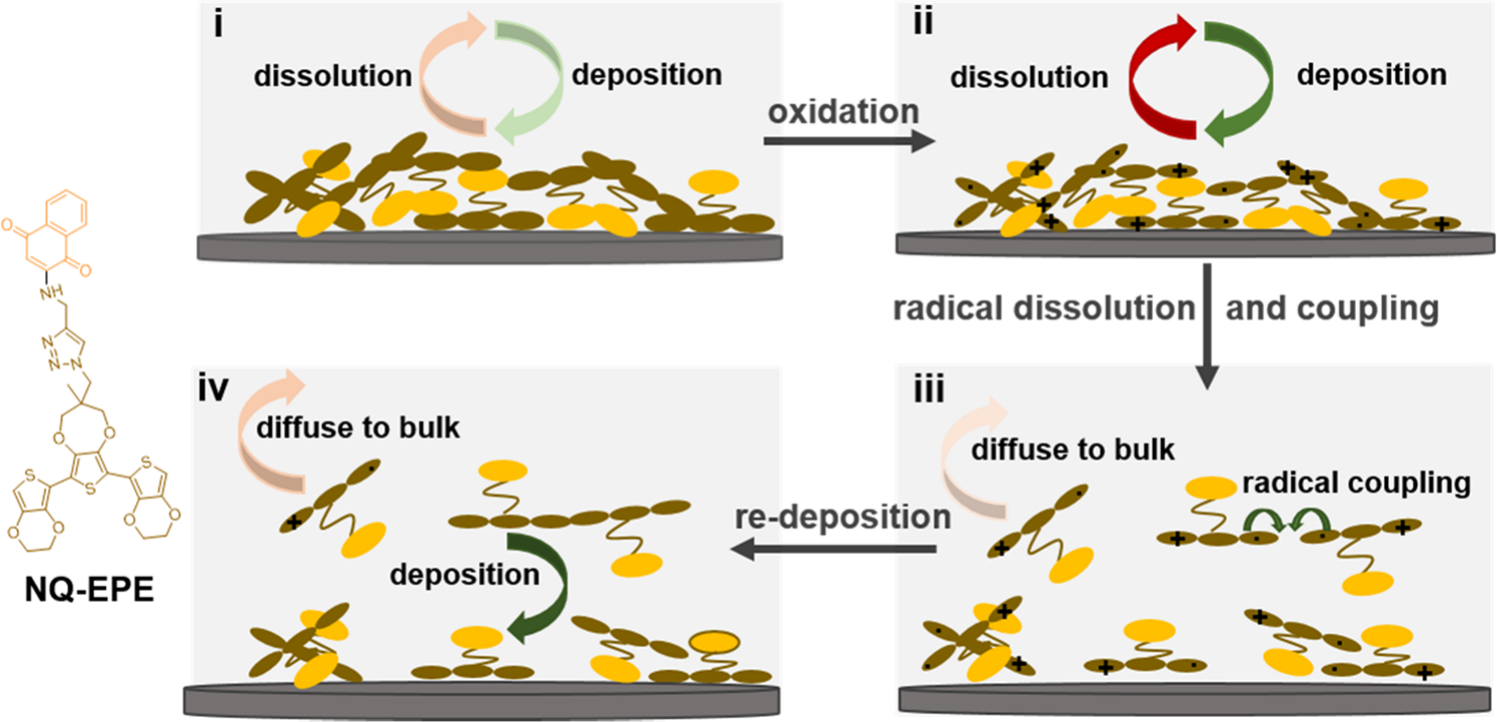
PDP Mechanism: (i) Glassy Carbon with Insoluble Neutral State Trimer Film in the Polymerization Solution, (ii) Formation of a Soluble Trimer Radical Cation upon Neutral State Trimer Oxidation, (iii) Competition between Trimer Radical Coupling and Diffusion to the Bulk Solution, and (iv) Oligomer Redeposition

We use thiophene-based ethynylpentiptycenylethynylene (EPE) as a repeating unit, while NQ and Qz, covalently attached to the central ProDOT unit, are acting as capacity-carrying components for the anode and cathode, respectively (Scheme 2). The trimer solution was drop-cast onto a glassy carbon current collector and vacuum-dried, forming a dark brown film. The glassy carbon electrode was then transferred to 0.1 M MeTriHTFSI/MeCN/H_2_O (vol MeCN: 67%) (Figure S14). No dissolution of the layer was observed prior to polymerization. Cyclic voltammetry (CV) was then used to polymerize the trimer layer in the above electrolyte during which the dark brown NQ-EPE trimer layer turned into a black, poly(NQ-EPE) film (Figure S15). In the first anodic CV scan, the current increases from 0.2 V (vs Fc^+/0^) (Figure 1a), which results from the oxidation of the neutral trimer. Oxidation of the trimer signifies the formation of trimer radical cations that can attack and couple to another trimer radical and form a hexamer. The hexamer can be oxidized further and couple to another radical, and the polymer grows. The observed trimer oxidation potential is much lower than that of the monomer (1.1 V vs Fc^+/0^),^42^ which is attributed to the extended aromatic system in the trimer.^43^ In the following three scans, polymerization continues with observed irreversible oxidation currents above 0.2 V (vs Fc^+/0^). During polymerization, a reversible, seemingly capacitive current, resulting from doping (oxidation) and dedoping (reduction) of the polymer backbone, builds up at potentials below 0.2 V (vs Fc^+/0^). Polymerization is completed in five scans, and only the rectangular-shaped capacitive current is observed between −0.1 and 0.6 V (vs Fc^+/0^). Polymerization of QzH_2_-EPE shows similar behavior to NQ-EPE except that the Qz/QzH_2_ redox peak centered at 0.4 V is observed and the peak current also builds up upon polymerization (Figure S16). IR spectroscopy shows that the characteristic vibrational peaks of quinone and trimer backbone were preserved after polymerization (Figure S26). The length of the resulting polymers was estimated from the charge consumed during polymerization (Figures S21 and S22). For poly(NQ-EPE) and poly(QzH_2_-EPE), the average polymer lengths were calculated to be 14 and 9 thiophene units, which are comparable to what is commonly found for polymers derived from traditional chemical and electrochemical polymerization methods.^44,45^ The estimated average molecular weights were estimated to be 3.35 kg/mol for poly(NQ-EPE) and 2.46 kg/mol for poly(QzH_2_-EPE).

*In situ* EQCM measurements revealed an extensive mass increase above 0.07 V (vs Fc^+/0^) during polymerization (Figure 1b). Both polymerization-induced solvent uptake and doping-induced uptake of charge-balancing bis(trifluoromethane)sulfonimide (TFSI) anions are associated with the mass increase. Assuming that the uptake of TFSI is reversibly expelled during the following cathodic scan, the solvent uptake can be estimated by the net mass change after the complete first cycle. Such estimation suggests 30 and 49 wt % solvent uptake for NQ-EPE and QzH_2_-EPE, respectively (Figure S17). The PDP process is thus associated with substantial swelling during polymerization.

*In situ* conductance measurements show that the conductance starts to increase from 0.1 V (vs Fc^+/0^) during the first anodic scan (Figure 1c). Polymerization of trimers produces polymer chains with increased conductance as a result. The conductance increases steadily even after scan reversal until 0.2 V (vs Fc^+/0^) due to the continuous polymerization at sufficiently high potentials, as confirmed by the positive currents observed during cathodic polarization. During the anodic sweep in the second scan, the conductance initially increases, as the obtained polymer is doped. However, the conductance reaches a maximum value of 0.9 mS at 0.21 V (vs Fc^+/0^) and decreases as the potential increases further (Figure 1d). The potential where the conductance reaches its maximum value is denoted *E*_max_^G^. As polymerization proceeds in consecutive scans, the overall conductance increases, *E*_max_^G^ shifts toward higher potentials, and the conductance peak becomes less and less pronounced (Figure S18). These features are well accounted for by the conversion of trimeric units to successively longer chains. When the chains are short, charge carriers are localized and electron transport is dominated by redox hopping between localized states. The conductance maximizes when half of the states are populated, i.e., at the average formal potential of the oligomers.^46−48^ As the polymer length increases, the increased delocalization of charges increases the interaction between successively induced charges and eventually leads to the continuous doping over an extended potential region observed for most conducting polymers. The broadening of the doping potential window is manifested in the appearance of a conductance plateau seen in the seventh scan (Figure S18f). Conductance monitoring during QzH_2_-EPE polymerization shows similar behavior to that in NQ-EPE except that the conductance peak does not evolve into a conductance plateau upon repeated cycling, indicating that the PDP process results in shorter chains of poly(QzH_2_-EPE) than those of poly(NQ-EPE) in accordance with the estimated polymer length (Supporting Information, SI, Section S4).

### 2.2 PDP Solution Optimization

Traditional electropolymerization from a monomer solution relies on the encounter of two radical cations formed upon oxidation in the vicinity of the electrode surface and their coupling. The polymer length is largely determined by the polymer solubility as chain propagation is terminated by precipitation.^49^ In the PDP method, however, the neutral trimers should not dissolve in the polymerization solution, which is a prerequisite for the method to work. One might therefore expect that chain propagation should be impossible since the termination condition is already met from start. We attribute the successful PDP above to partial dissolution of the intermediate radical cation formed upon oxidation (Scheme 2, stage ii). This would allow the encounter and coupling of trimer radicals to form longer chains. There should hence be a balance between favorable dissolution of the intermediate that allows for chain propagation and detrimental dissolution of the neutral trimer causing loss of the material.

**Figure 2:**
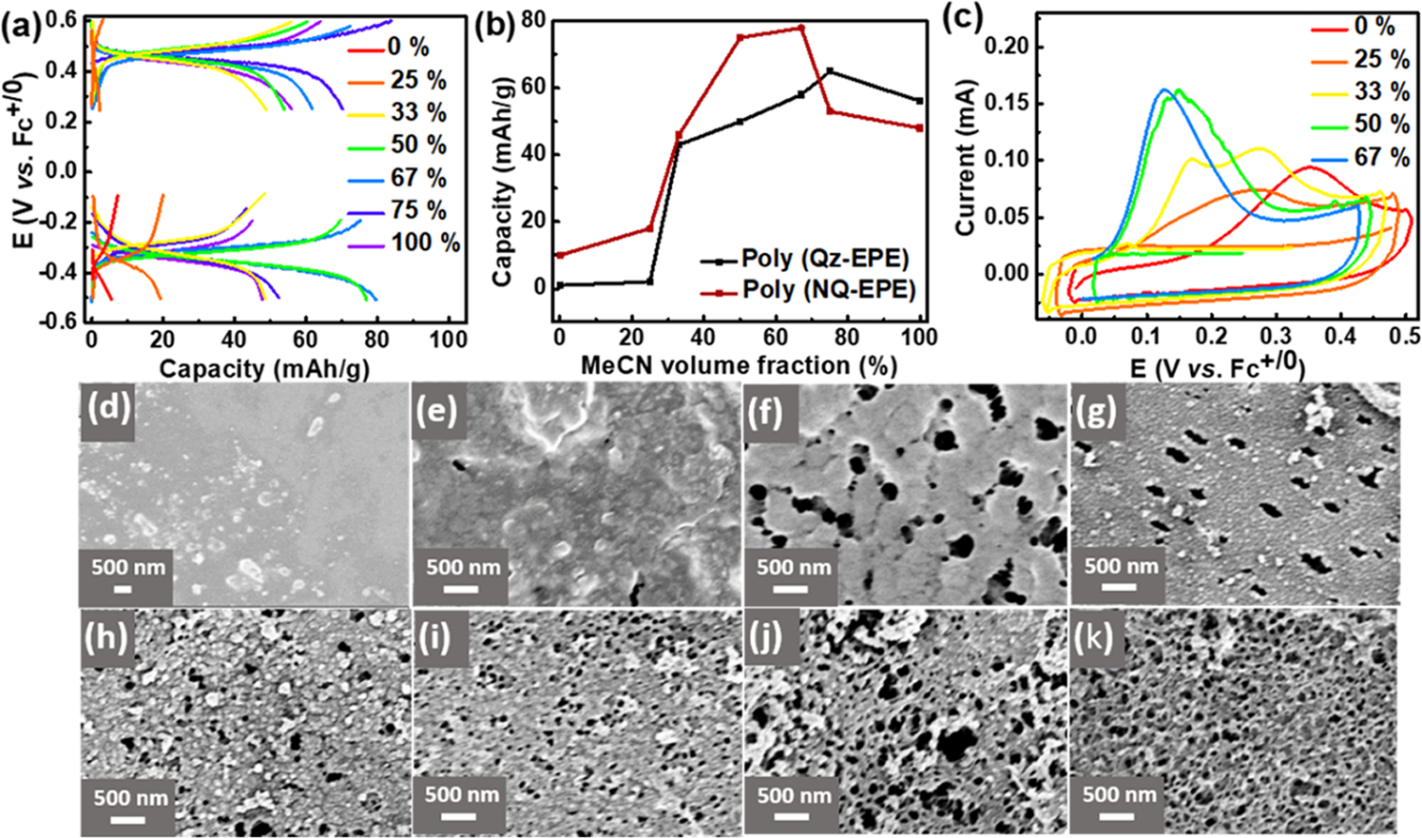
(a) Galvanostatic charge–discharge curves of 0.1 mg of poly(QzH_2_-EPE) (upper) and 0.1 mg of poly(NQ-EPE) (bottom) tested in a three-electrolyte setup in the MeTriHTFSI electrolyte at a current density of 0.3 A/g. Different colors represent polymers polymerized in 0.1 M MeTriHTFSI/MeCN/H_2_O solution with different MeCN volume fractions. (b) Corresponding discharge capacity of the two polymers as a function of the MeCN volume fraction in the polymerization solution. (c) Polymerization cyclic voltammograms of 10 μg of NQ-EPE at 8 mV/s in 0.1 M MeTriHTFSI/MeCN/H_2_O with different MeCN volume fractions. SEM micrographs of the NQ-EPE trimer film (d) and poly(NQ-EPE) polymerized in (e) 0%, (f) 25%, (g) 33%, (h) 50%, (i) 67%, (j) 75%, and (k) 100% MeCN volume fraction in 0.1 M MeTriHTFSI/MeCN/H_2_O. All experiments were conducted using a glassy carbon current collector.

To investigate the balance between the favorable dissolution for chain propagation and detrimental dissolution of trimer loss, a series of experiments where the solvent was systematically varied were conducted. The two neutral trimers, QzH_2_-EPE and NQ-EPE, both dissolve in pure MeCN, while neither dissolve in H_2_O. MeCN thus causes detrimental dissolution of the neutral trimer and, at the same time, favorable dissolution of the intermediate required for chain propagation. By the addition of H_2_O, the solubility can be reduced, and by controlling the amount of H_2_O, the balance between the two effects can be optimized. To find the optimum solvent composition, a series of polymerization solutions of 0.1 M MeTriHTFSI/MeCN/H_2_O with different MeCN volume fractions were used and the resulting polymers were characterized in MeTriHTFSI. Figure 2a shows the charge–discharge profiles of poly(QzH_2_-EPE) (upper curves) and poly(NQ-EPE) (lower curves) in the MeTriHTFSI electrolyte. Poly(QzH_2_-EPE) exhibited a discharge plateau centered at 0.45 V (vs Fc^+/0^), which is attributed to Qz/QzH_2_ redox conversion (Scheme 1), while the discharge plateau of poly(NQ-EPE) was centered at −0.35 V (vs Fc^+/0^), which is attributed to NQ/NQH_2_ redox conversion (Scheme 1). The discharge capacity of the two polymers shows a clear dependence on the MeCN volume fraction in the polymerization solution (Figure 2b) with the plateau capacity reaching the maximum at 67 and 75% for poly(NQ-EPE) (78 mAh/g) and poly(QzH_2_-EPE) (68 mAh/g), respectively. The obtained maximum capacities reached the theoretical capacity of corresponding poly(NQ-EPE) (78 mAh/g) and poly(QzH_2_-EPE) (68 mAh/g), respectively. We attribute the decrease in capacity observed at higher MeCN concentrations to dissolution loss of the neutral state trimer, which was already observed prior to polymerization in these concentrations. For the increase in capacity with increased MeCN content, we hypothesize that this is related to the increased rearrangement of the radical intermediate that comes with the increased solubility in the electrolyte.

To test the hypothesis of the rearrangement of the radical intermediate, the resulting polymers were further examined. Figure 2d shows that prior to polymerization the NQ-EPE trimer film is solid and smooth with almost no microstructure. Polymerization of NQ-EPE caused significant morphological changes indicating that the trimers indeed do rearrange during the polymerization process. In addition, the polymers become increasingly rough and porous (Figure 2e–i) with an increased MeCN fraction in the polymerization solution, indicating that MeCN facilitated the rearrangement of the radical intermediate. With even higher MeCN volume fractions, the polymer layer transforms to a highly porous network of ∼60 nm thick interconnected nanowires (Figure 2j,k). Polymerization of QzH_2_-EPE also caused morphological changes to the layer. However, a MeCN volume fraction of more than 33% was needed to induce any significant morphology changes as polymerizations using lower MeCN contents gave morphologies that are inseparable from the initial trimer layer (Figure S20).

The MeCN content also affects the polymer length: in 0% MeCN, the average poly(NQ-EPE) was six thiophene units, while the average polymer length was 14 thiophene units in 67% MeCN (Table S1). The lower rearrangement ability of QzH_2_-EPE was also reflected in shorter polymer length, and we estimate the average polymer length to be four and nine thiophene units for poly(QzH_2_-EPE) formed with 0% MeCN and 75% MeCN in the polymerization solution, respectively (Table S2). We also noticed that the short chains subsequently polymerized during electrochemical characterization in the MeTriHTFSI electrolyte (Figures S23 and S24).

As stated above, we speculate that the rearrangements observed are related to rearrangements of the radical intermediates and the polymerization voltammograms provide some support for this hypothesis (Figure 2c (NQ-EPE) and Figure S25 (QzH_2_-EPE)). The irreversible anodic peaks correspond to oxidation of neutral trimers to the radical intermediates that ultimately lead to the coupling of trimer segments. With increased MeCN content in the polymerization solution, the oxidation peak shifts towards lower potentials, suggesting stabilization of the oxidation products or destabilization of the reactants. As the reactants in all cases are insoluble neutral trimers irrespective of the polymerization electrolyte, the latter possibility is unlikely. If the oxidation products, i.e., the charged radical intermediates, become increasingly soluble as the MeCN content increases, the energy for the oxidation products would be stabilized by the same mechanism. Hence, the shift in peak position could be accounted for by the transient dissolution of the oxidation products (stage ii, Scheme 2). Due to the subsequent radical coupling and deprotonation, the soluble radical intermediates are prevented from leaving the electrode surface and reprecipitate instead (stage iv, Scheme 2).

In summary, rearrangements of the deposited trimer layer during PDP are required to reach the full charge storage capacity of the materials. Such rearrangement requires transient dissolution of the material. Dissolution of the initial reactants, however, would lead to the loss of active materials, and hence, rearrangements must be accomplished by intermediate species formed during polymerization instead. Finite solubility of one or more of the reaction intermediates can be accomplished by judicious choice of the solvent. We thus propose the polymerization mechanism for PDP that includes (1) oxidation of the neutral trimer, (2) dissolution of radical cations, (3) radical–radical coupling, and (4) redeposition of oligomers. The obtained maximum capacity from the optimized polymerization solution is close to the theoretical capacity, and all of the characterized polymers below (poly(NQ-EPE) *M*_w_: 3.35 kg/mol and poly(QzH_2_-EPE) *M*_w_: 2.46 kg/mol) are obtained under optimized polymerization conditions, namely, 0.1 M MeTriHTFSI/MeCN/H_2_O (vol MeCN: 67% for NQ-EPE and vol MeCN: 75% for QzH_2_-EPE).

### 2.3 Characterization of Individual Electrodes

**Figure 3:**
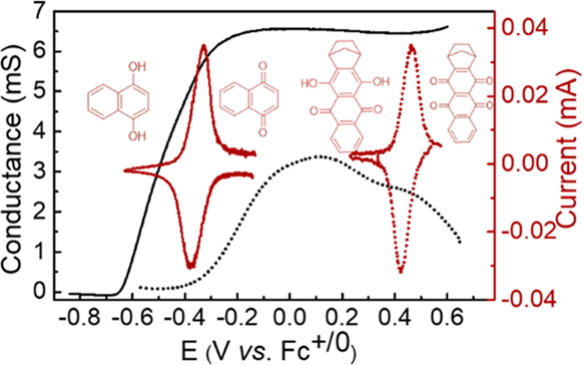
*In situ* conductance (black) on IDA Au electrodes in 0.1 M MeTriHTFSI/MeCN and cyclic voltammograms (red brown) on glassy carbon electrodes in MeTriHTFSI of poly(NQ-EPE) (solid line) and poly(QzH_2_-EPE) (dotted line) as a function of the potential at a scan rate of 0.1 mV/s.

*In situ* conductance measurements were used to monitor the dependence of polymer conductance on the applied potential. The results show that appreciable conductance is observed at potentials above −0.65 V (vs Fc^+/0^) and −0.38 V (vs Fc^+/0^) for poly(NQ-EPE) and poly(QzH_2_-EPE), respectively (Figure 3). The increase of conductance is related to the oxidation (or doping) of the polymer backbone: upon oxidation, the polymer backbone becomes positively charged and the formed radical cation (polaron) is mobile and acts as a charge carrier. With the formation of charge carriers upon oxidation of the polymer backbone, the conductance of the material therefore increases rapidly over a narrow potential region. The conductance of poly(NQ-EPE) reached a rather constant value (∼6 mS) at potentials above −0.2 V (vs Fc^+/0^). This is a common feature of conducting polymers that is related to continuous doping over a wide potential region.^50,51^ Poly(QzH_2_-EPE) shows a markedly different dependence of the conductance on potential with a clear maximum of 3.4 mS, centered at 0.1 V (vs Fc^+/0^). Such behavior has more resemblance to redox hopping between redox sites, and we hypothesize that this can be traced to the short polymer chains in poly(QzH_2_-EPE) since shorter chains would give a narrower potential region where the polymer is redox-active. Irrespective of the transport mechanism, the conductance provided by the polymer backbone was found to be sufficient to allow efficient electron transport pathways for the pendant group redox conversion without using any conducting additives.

Poly(NQ-EPE) and poly(QzH_2_-EPE) show one pair of redox peaks centered at −0.35 V (vs Fc^+/0^) and 0.45 V (vs Fc^+/0^), respectively. The two redox reactions are assigned to the two-electron redox conversion of the respective pendant group that, in both cases, is assumed to be coupled to the transfer of two charge-balancing protons (Scheme 1). The ratios between the anodic and cathodic peak currents are unity (*i*_p(red)_/*i*_p(ox)_ = 1), suggesting that both of the pendant group redox conversions are chemically reversible. Although the anodic and cathodic peak potentials do not coincide even at the lowest scan rate used (0.1 mV/s), the Gaussian peak shape suggests that the redox reactions appear as surface-confined redox processes (Figures S28 and 29). At scan rates above 1 mV/s, the peaks show significant broadening and increased separation between the anodic and cathodic peaks. However, integration of the redox peaks shows full conversion of the layer below 10 mV/s, suggesting that the reaction is not limited by mass transport but rather by limited electron transfer rates or resistance. Poly(QzH_2_-EPE) exhibited a second pair of redox peaks centered at −0.4 V (vs Fc^+/0^) corresponding to QzH_2_/QzH_4_ redox transfer (Figure S30). Compared to the Qz/QzH_2_ peak, the QzH_2_/QzH_4_ peak is much smaller, which is likely an effect of redox mismatch between the polymer backbone and the QzH_2_/QzH_4_ redox reaction. The redox mismatch for the second reduction leads to incomplete redox conversion and, hence, a low capacity for the redox reaction (Figure S30).

To confirm the peak assignment above, the intensity change of the IR absorption from characteristic carbonyl vibrational peaks upon reduction/oxidation was monitored. Upon oxidation of poly(QzH_2_-EPE), the absorption peak at 1631 cm^–1^, attributed to the stretching of the Qz-carbonyl group (−C=O),^52^ strengthened (Figure S27b), indicating that Qz experienced a transition from the benzoid structure to the quinoid structure. In poly(NQ-EPE), the absorption peak at 1627 cm^–1^, which is assigned to the stretching vibration of the carbonyl group on NQ, weakened upon the reduction (Figure S27a) in agreement with the assignment of the peak to the reduction of NQ.

Individual characterization of poly(QzH_2_-EPE) and poly(NQ-EPE) produced by PDP thus shows that both polymers are electrically conductive. The conductance is traced to the EPE backbone, and, as for all conducting polymers, ground-state conductivity requires that the polymer backbone is charged or doped. Furthermore, in MeTriHTFSI, both the NQ/NQH_2_ and Qz/QzH_2_ redox conversions are redox-matched with the polymer backbone and the two redox reactions are reversible with capacities close to the theoretical capacity. In contrast, the nonredox matched QzH_2_/QzH_4_ reaction shows capacities far below the theoretical capacity, stressing the importance of redox matching in the CRP design. In CRPs, the dominant capacity is carried by the redox conversion of the pendant groups occurring at −0.35 V (vs Fc^+/0^) for poly(NQ-EPE) and 0.45 V (vs Fc^+/0^) for poly(QzH_2_-EPE), respectively. Combining the two materials into battery cells with poly(QzH_2_-EPE) as the cathode and poly(NQ-EPE) as the anode should hence give a secondary battery with an average voltage output of 0.8 V and a reversible capacity close to the theoretical capacity of the polymers. We therefore decided to combine the two battery materials into complete battery cells using MeTriHTFSI as the electrolyte.

## 3 Battery Performance

Some batteries were fabricated with the cathode as the limiting electrode and some with the anode as the limiting electrode to enable separate evaluation of the two materials in a battery configuration, where an 0.8 V output is expected. During charging, QzH_2_ is oxidized to Qz and releases two protons, while NQ takes up two protons upon reduction to NQH_2_ (Scheme 1); the opposite reactions occur during discharge. The dominant process during charge and discharge can thus be described as a proton rocking-chair motion with proton flux toward the anode during charge and opposite flux during discharge.

**Figure 4:**
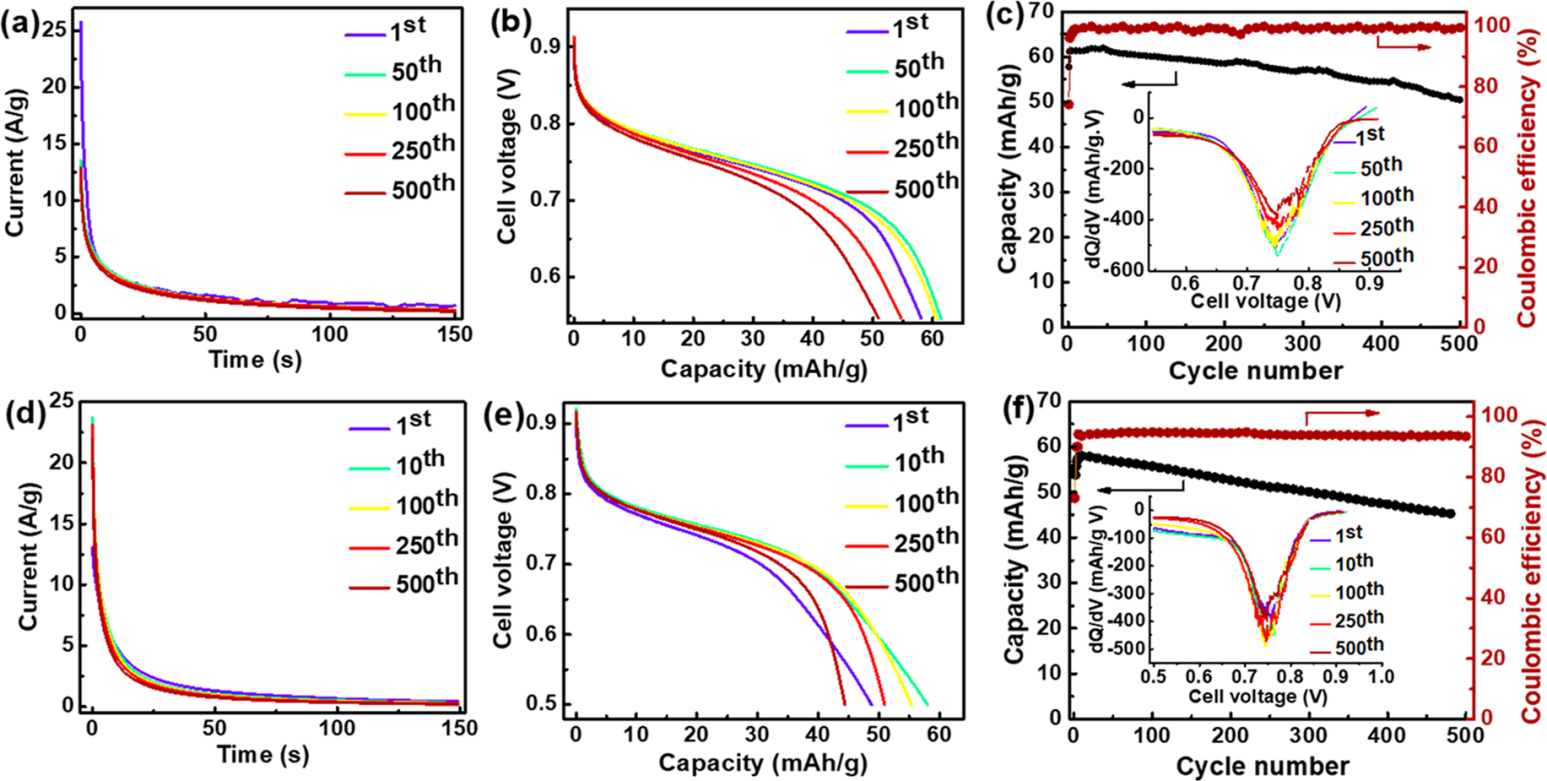
Battery performance using poly(NQ-EPE) as the limiting material (upper panels) and poly(QzH_2_-EPE) as the limiting material (bottom panels) with MeTriHTFSI as the electrolyte. Current response as a function of time during the constant voltage charging at 1 V (a) and 0.95 V (d). Discharge profile of different cycles at a current density of 0.3 A/g (b, e). Corresponding stability and Coulombic efficiency (c, f); the inset figures show differential plots of the discharge curve (d*Q*/d*V*) against voltage.

Galvanostatic charge–discharge showed that the assembled battery suffered from high internal resistance, resulting in incomplete charging at the cutoff potential (Figure S31). The potential step charging method was thus used to enable fast, full charging as well as suppress side reactions.^53^ With poly(NQ-EPE) as the limiting material (Figure 4, upper panel), a charging voltage of 1 V was applied, which resulted in an initial charging current of 26 A/g (Figure 4a). Within 27 s, half of the charging process was completed and full charge was achieved within 150 s (Figure 4a). In batteries with poly(QzH_2_-EPE) as the limiting material, a slightly lower charging voltage (0.95 V) was used to avoid irreversible oxidation of the electrolyte (Figure S32). In this case, the initial current density was 24 A/g (Figure 4d).

Galvanostatic discharge of both battery types gave voltage plateaus centered at around 0.8 V (Figure 4b,e). Distinct peaks in d*Q*/d*V*-plots indicated that the highest capacity was observed at 0.78 V (insets, Figure 4c,f), corresponding well to the difference in formal potential between the NQ/NQH_2_ and the Qz/QzH_2_ redox conversion (Figure 3). A significant residual capacity below 0.65 V was also observed in the discharge curve due to the polymer backbone, which is expected to cycle between doping levels 0.1 and 0.2 for the anode in the anode-limiting case and between 0.5 and 0.7 for the cathode in the cathode-limiting case. With poly(NQ-EPE) as the limiting material, the discharge capacity at a current density of 0.3 A/g (4.5 C) was 62 mAh/g, as evaluated from the discharge capacity normalized to the mass of the limiting electrode, which is about 80% of the theoretical capacity of the NQ/NQH_2_ redox conversion (78 mAh/g). The discharge capacity increased gradually during the first 50 cycles probably as a result of increased swelling of the polymer during cycling. Polymer swelling was supported by EQCM measurements as a continuous mass increase upon cycling in MeTriHTFSI was observed (Figure S34). The anode-limiting battery retained 80% of the highest observed capacity after 500 cycles (Figure 4c), and the peak positions observed in the d*Q*/d*V* plot were well preserved, indicating that the nonlimiting cathode was sufficiently stable as to provide a stable reference potential. The close to 100% Coulombic efficiency also suggests that no (or only minor) irreversible side reactions occurred in the anode-limiting device. This 0.8 V battery was also proved to be able to power a red light-emitting diode by utilizing three batteries in series (Figure S35). The cathode-limiting device showed a similar initial discharge capacity (58 mAh/g) to the anode-limiting device at a current density of 0.3 A/g (4.5 C), corresponding to 85% of the theoretical capacity of the Qz/QzH_2_ redox conversion (68 mAh/g). The Coulombic efficiency was, however, much lower (∼95%), and 75% of the capacity was retained after 500 cycles.

**Figure 5:**
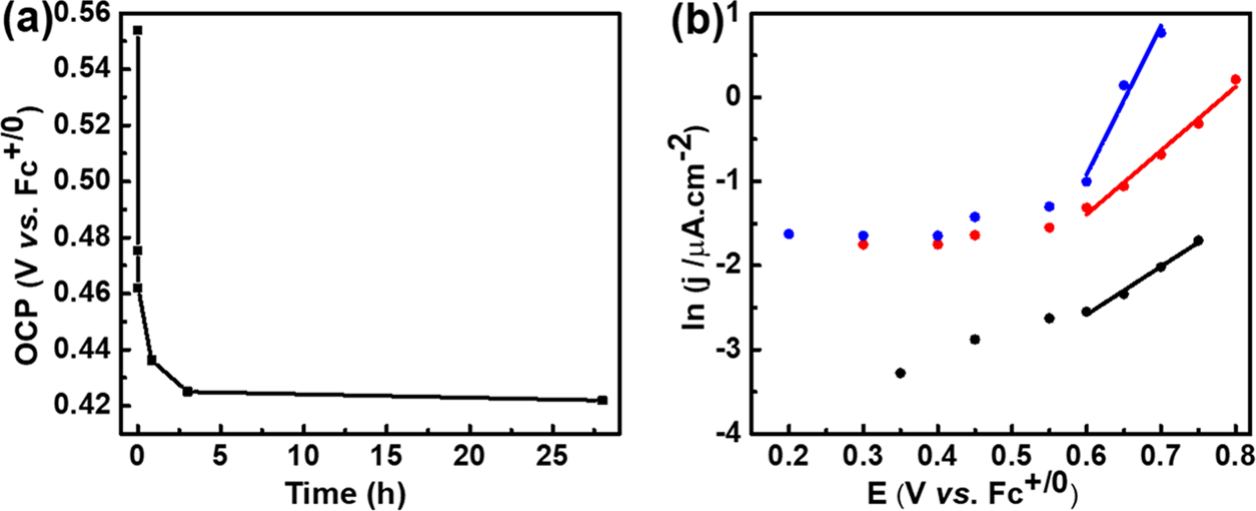
(a) Open-circuit potential relaxation of poly(QzH_2_-EPE) in a three-electrode setup after potentiostatic charging at 0.7 V for 150 s. (b) Plots of leakage current of bare glassy carbon (black), poly(EPE) on glassy carbon (red), and poly(QzH_2_-EPE) on glassy carbon (blue) as a function of applied potential in MeTriHTFSI.

To investigate the origin of the low Coulombic efficiency of the cathode-limited device, self-discharge of the charged cathode was investigated. The poly(QzH_2_-EPE) electrode was first charged by applying a constant potential of 0.7 V (vs Fc^+/0^) for 150 s in a three-electrode setup. The open-circuit potential (OCP) was then monitored during a fixed period of time after which the electrode was galvanostatically discharged, the remaining capacity was recorded, and the electrode was then recharged, and the cycle was repeated. Figure 5a shows that the OCP decayed to 0.43 V (vs Fc^+/0^) within 3 h and 33% of the capacity was lost (Figure S36). During the next 25 h, the OCP slowly dropped to 0.42 V (vs Fc^+/0^) and an additional loss in capacity of 13% was observed (Figure S36). In an attempt to determine the mechanism of charge loss in the cathode, leakage current experiments were conducted. A fixed potential was applied, and the residual current was measured at various potentials (Figure S37). In all cases, the leakage current increased exponentially with potential above 0.6 V (vs Fc^+/0^) (Figure 5b), suggesting that the leakage current in all cases was kinetically limited by a redox reaction. The bare electrode also showed exponential dependence of the leakage current on potential, suggesting that the electrolyte MeTriHTFSI undergoes irreversible oxidation that, in principle, could partially account for the charge losses. However, the leakage currents on polymer-covered electrodes are much higher than that on bare glassy carbon, which could be due to the larger surface area in these electrodes covered by the polymer. However, it is unlikely that the increased surface area could account for the order-of-magnitude increase in leakage current. A more likely explanation is that the irreversible oxidation reaction of the polymer dominates the current leakage. As the poly(QzH_2_-EPE)- and the poly(EPE)-covered electrodes show similar responses, it is likely that the reaction is related to the polymer backbone rather than the Qz. Overoxidation is a feature for conducting polymers that could be the origin of the leakage current,^54−57^ but we cannot exclude the possibility that the polymer-centered redox reaction is triggered by the degradation of the electrolyte at this stage. We thus conclude that the low Coulombic efficiency in the cathode-limiting cell is due to irreversible redox reactions of the polymer backbone. The strong dependence of this reaction on potential makes it possible to suppress the charge loss and improve the Coulombic efficiency by avoiding complete oxidation of the cathode, as was demonstrated in the anode-limiting device.

## 4 Conclusions

In this report, we have successfully produced CRP films by PDP. The use of trimers allows for polymerization under mild conditions, and the PDP method allows for 100% utilization of the starting materials. We have shown that successful PDP relies on the transient dissolution and rearrangement of the intermediate radical cation state during polymerization that can be controlled by the solvent composition of the polymerization electrolyte. The choice of polymerization solvent composition can tune the polymer properties, and it strongly affects polymer morphologies, polymer lengths, and the electrochemical properties of the resulting polymer. The polymer conductance enabled the use of the materials as active electrode materials directly, without the need for any conducting additives. By utilizing two different pendants, NQ and Qz, a potential difference of 0.8 V was achieved in the protic ionic liquid electrolyte. By combining the two materials, an all-organic proton rocking-chair battery with a protic ionic liquid electrolyte was produced and tested.

## Supporting Information Available

Synthesis and characterization of monomers with NMR data; supplemental experimental information during PDP, including, CV, *in situ* conductance and EQCM; and polymer characterization, including SEM images, polymer length calculation, *in situ* and *ex situ* FTIR, kinetic study, EQCM, self-discharge, and leakage current (PDF)
