# Supplementary material for: Rocking-Chair Proton Batteries with Conducting Redox Polymer Active Materials and Protic Ionic Liquid Electrolytes
Source: ACS Appl Mater Interfaces. 2021 Apr 15;13(16):19099–108. doi: 10.1021/acsami.1c01353 (PMC8153541; doi:10.1021/acsami.1c01353)
Supplement: Supplementary file 1 — am1c01353_si_001.pdf [file am1c01353_si_001.pdf]

Supporting Information

Rocking-Chair Proton Batteries with Conducting Redox Polymer Active Materials and Protic Ionic Liquid Electrolytes

Huan Wang[†], Rikard Emanuelsson[,†], Christoffer Karlsson[‡], Patric Jannasch[‡], Maria Strømme[†], Martin Sjödin[**,†]*

[†] Nanotechnology and Functional Materials, Department of Materials Science and Engineering, The Ångström Laboratory, Uppsala

University, P.O. Box 35, SE-751 03, Uppsala, Sweden

[‡] Centre for Analysis and Synthesis, Department of Chemistry, Lund University, P.O. Box 124, SE-221 00 Lund, Sweden

[*] E-mail: rikard.emanuelsson@angstrom.uu.se.

[**] E-mail: martin.sjodin@angstrom.uu.se.

Table of Contents

Section 1: Synthesis....3
S1: General synthetic information....6
S1: Instrumentation....6
S1: Synthesis procedure details....7
Section 2: Experimental procedures....27
S2: Electrode preparation....27
S2: Electrochemical characterization....28
S2: Battery evaluation....29
S2: Other characterization....30
Section 3: Post-deposition polymerization (PDP)....30
S3: Cyclic voltammogram....31
S3: Solvent-uptake in *in situ* EQCM measurement....32
S3: Conductance builds up in *in situ* conductance measurement....33
Section 4: Polymerization solution optimization....35
S4: Polymer morphology evolution....35
S4: Polymer length....35
S4: oxidation potential of neutral state trimer....40
Section 5: Polymer characterization....42
S5: *ex situ* ATR and *in situ* FTIR....42
S5: kinetic study....43
S5: redox match....45
Section 6: Battery evaluation....46
S6: *in situ* EQCM measurement....47
S6: self-discharge....49
S6: leakage current....50
Reference....51

Section 1: Synthesis

The starting point for this study is the terthiophene unit we have recently synthesized and functionalized. Here, we show further that the alcohol terthiophene [**EP(OH)E**] is a versatile handle which can be used to attach an terminal alkyne unit suitable for *i.e.* click or Sonogashira chemistry [**EP(CCH)E]** and, via the meyslated unit [[**EP(OMs)E**], transformed to an azide [**EP(N_3)E]** suitable for the orthogonal click chemistry. We were interested in these more robust linkers as our previous linkers, an ester and a thioether, used to attach the terthiopene to the redox active unit could be susceptible to cleavage under the conditions used in batteries. **EP(N_3)E** could be smoothly constructed via substitution of the mesylate using a mixture of NaN_3 and NaI in dimethyl sulfoxide (DMSO) to give the azide. Alkylation of **EP(OH)E** with propargyl bromide required a biphasic system (NaOH in water/toluene) and a phase transfer catalyst. A small amount of acetone was used to ensure **EP(OH)E** dissolved properly in the toluene layer. After stirring for 3 days **EP(CCH)E** was isolated in good yield.

Quinizarin (Qz) is an interesting redox-active compound for organic energy storage due to its high redox potential, a consequence of the strongly electron withdrawing central benzoquinone unit. However, the oxidized form is susceptible to addition reactions both over the central and terminal double bonds. To protect the terminal bonds we utilized a fused norbornane unit which also provides increased solubility compared to *i.e.* methyl substituents. Reacting 3,6-dihydroxybenzonorbornane, a commercially available hydroquinone with a fused norbornane moiety, with 4-bromophthalic acid in a $AlCl_3$/NaCl melt gave Qz **1**. The phenolic alcohols were then acetyl protected to prepare it for subsequent chemistry giving **2**. The acetyl protected **2** can be easily purified from iPrOH and thus we did not optimize any recrystallization conditions for **1**. Sonogashira coupling between **2** and **EP(CCH)E** using a microwave reactor connected Qz to the terthiophene. Deprotection of the acetyl groups is easily achieved by stirring the compound in

a mixture of diethylamine and dichloromethane (DCM). The resulting *N*,*N*-diethylacetamide formed is removed during workup to give pure **QzH2-EPE**. For the negative electrode we synthesized a naphthoquinone (NQ) substituted with an amine to create an analogue to our previously reported system. Using propargyl amine the amino group was smoothly added to the NQ double bond while providing a terminal alkyne suitable for further chemistry (**3**). Heating **3** and **EP(N3)E** with 20 mol% CuI in dimethylformamide (DMF) at 100 °C gave the desired **NQ-EPE.**

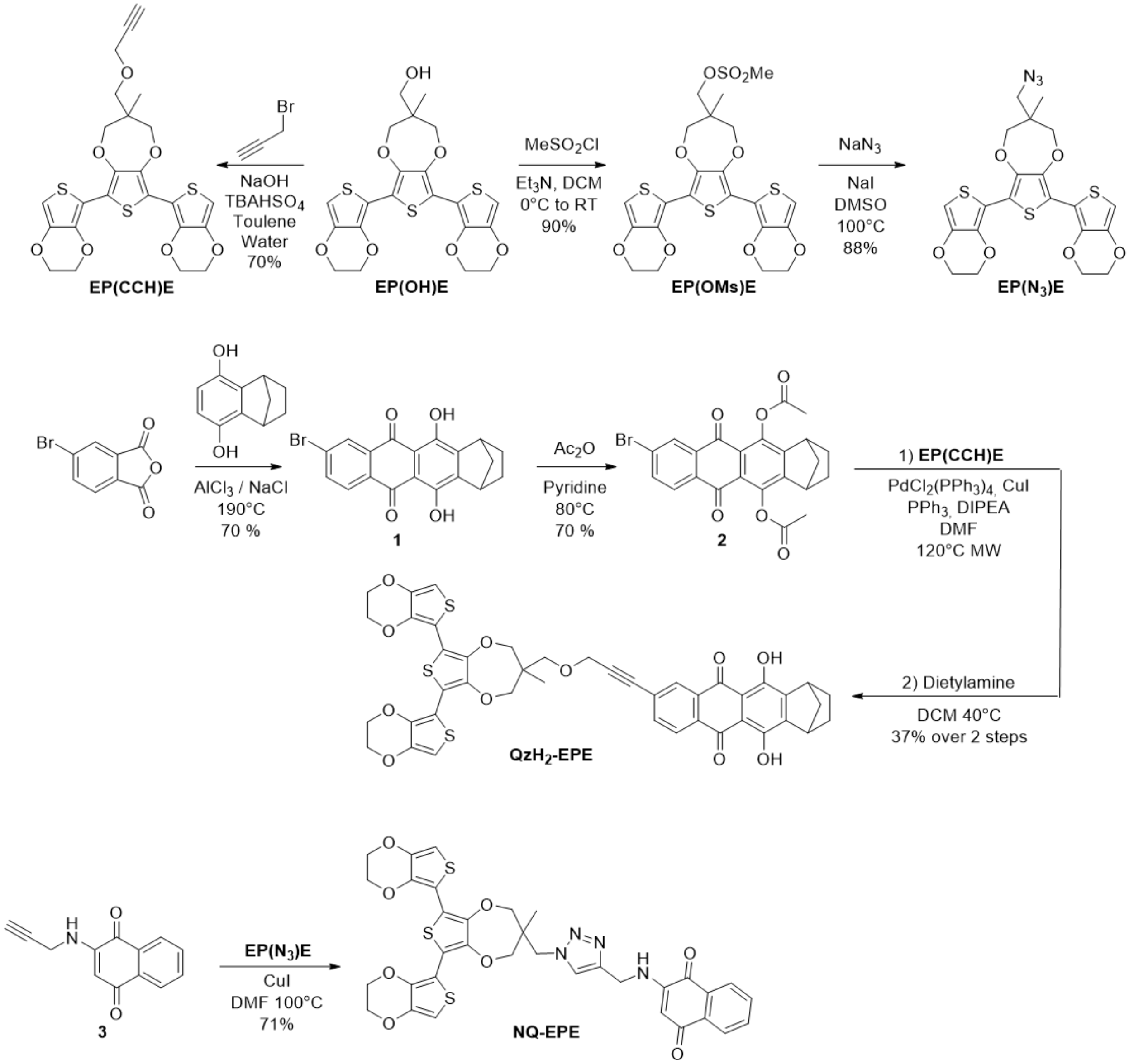

Figure S1. Overview of the synthesis of functionalized terthiopenes, capacity carrying redox active units and their subsequent attachments.

S1: General synthetic information

All reactions were performed in flame-dried glassware under an argon atmosphere unless otherwise stated. Room temperature (RT) refers to 22 °C. Reagents were purchased commercially and used without further purification. DCM was dried by passing the solvent through activated aluminum using a PureSolv PS - MD - 4 - EN solvent purification system. Anhydrous DMF and DMSO were obtained from Sigma-Aldrich/Merck. Analytical thin layer chromatography was performed using pre-coated Merck Silica 60 F254 plates. Compound visualization was achieved with UV light (254 nm).Trimeric compounds darken under prolonged exposure to UV light and require no extra visualization aid, other compounds were visualized using potassium permanganate stain and heated when needed. Flash chromatography was performed on a Grace REVELERIS® X2 flash chromatography system using pre-packed silica cartridges (12 or 40 g, 40-63 μm). Dry loading was used in all cases and the crude was loaded onto silica using a suitable solvent which was subsequently removed under vacuum. The silica was then packed in a dry loading cartridge and eluted using the specified gradient and solvent combination. **EP(OH)E**, **EP(OMs)E**[1] and **3**[2] were prepared as previously described and their spectroscopic data was in agreement with reported data.

S1: Instrumentation

Nuclear magnetic resonance (NMR) spectroscopy: NMR spectras were recorded using an Agilent 400-MR (^{1}H at 400 MHz, ^{13}C at 101 MHz) equipped with a OneNMR probe, or using a Bruker Avance Neo (^{1}H at 500 MHz, ^{13}C at 126 MHz) equipped with a TXO (CRPHe TR-^{13}C/^{15}N/^{1}H 5 mm-Z) cryoprobe. Chemical shifts are reported using the residual solvent signal as an indirect reference to TMS ($CHCl_3$: δ_H = 7.26 ppm, δ_C = 77.16 ppm). Coupling constants (*J*) are reported in Hz and the following abbreviations (or combination thereof) were used to explain multiplicities: s = singlet, d = doublet, t = triplet q = quartet, m = multiplet, br = broad.

High resolution mass spectrometry: High-resolution mass spectra were acquired on a Waters LCT PREMIER operating in positive APCI or ESI mode. Samples were referenced against leucine-enkaphalin or sulfadimethoxine (depending on mass). MassLynx version 4.1 was used to analyze the results, this version of software does not account for the electron and all the calibrations/references are calculated accordingly.

S1: Synthesis procedure details

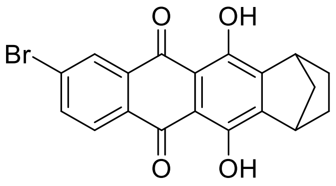

8-bromo-5,12-dihydroxy-1,2,3,4-tetrahydro-1,4-methanotetracene-6,11-dione (**1**)

4-bromophthalic acid (7.0 g, 28.6 mmol) and 3,6-dihydroxybenzonorbornane (5.0 g, 28.6 mmol) were mixed together and were added into a 500 mL round bottomed flask containing a 190 °C melt of $AlCl_3$ (40 g) and NaCl (10 g). Violent gas evolution occurred upon addition and the solution was stirred at 190 °C for 5 min. Thereafter the dark red mixture was cooled to 100 °C and ice was very carefully added until gas evolution ceased. The volume was adjusted to ~350 mL using water where HCl (30 mL, conc) was added. The red solution was refluxed for 2 h and filtered while it was hot. The red solid was washed with water and then dried under vacuum giving **1** as red powder of acceptable purity.

Yield: 8 g (70%), red powder

^{1}H NMR (500 MHz, $CDCl_3$) δ 12.99 (s, 1H), 12.91 (s, 1H), 8.39 (d, *J* = 1.9 Hz, 1H), 8.13 (d, *J* = 8.3 Hz, 1H), 7.88 (dd, *J* = 8.3, 1.9 Hz, 1H), 3.80 (m, 2H), 2.03 (m, 2H), 1.81 (m, 1H), 1.60 (m, 1H), 1.27 (m, 2H).

^{13}C NMR (126 MHz, $CDCl_3$) δ 186.2, 185.7, 153.2, 148.8, 148.6, 137.2, 134.9, 132.4, 129.93, 129.88, 128.6, 112.2, 112.1, 49.2, 40.8, 40.8, 25.7.

HRMS (APCI) calcd for $C_{19}H_{14}O_4Br^+$, M + H]$^+$: 385.0070; found: 385.0062

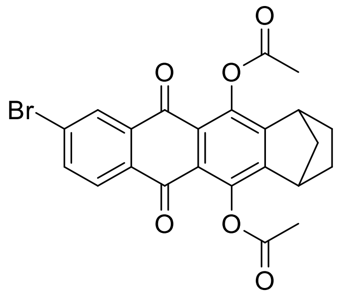

8-bromo-6,11-dioxo-1,2,3,4,6,11-hexahydro-1,4-methanotetracene-5,12-diyl diacetate (**2**)

1 (7.0 g, 18.1 mmol, 1 eq) was dissolved in a mixture of pyridine (15 mL) and acetic anhydride (35 mL). The solution was heated at 70 °C for 36 h. The solvents were removed under vacuum and repeatedly co-evaporated with toluene to remove all pyridine. The remaining brown solid was suspended in boiling iPrOH (200 mL) and sonicated. After cooling to RT the solution was placed in a refrigerator overnight. Then **2** was filtered off and dried under vacuum. The mother liquid was concentrated and the process was repeated (each time using less and less iPrOH) further three times to maximize the yield of **2**.

Yield: 6.0 g (70%), beige powder

^{1}H NMR (500 MHz, $CDCl_3$) δ 8.27 (s, 1H), 8.00 (d, J = 8.3 Hz, 1H), 7.83 (d, J = 8.3 Hz, 1H), 3.61 (m, 2H), 2.50 (s, 6H), 1.99 (m, 2H), 1.88 (m, 1H), 1.63 (m, 1H), 1.32 (m, 2H).

^{13}C NMR (126 MHz, $CDCl_3$) δ 181.6, 181.2, 169.5, 169.5, 150.5, 150.3, 141.7, 136.9, 134.6, 132.2, 129.8, 129.5, 128.6, 124.8, 48.8, 41.4, 25.7, 21.2.

HRMS (ESI) calcd for [$C_{23}H_{18}O_6Br^+$ + H, M + H]$^+$: 469.0280; found: 469.0286

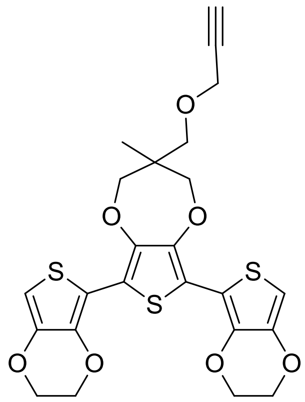

6,8-bis(2,3-dihydrothieno[3,4-b][1,4]dioxin-5-yl)-3-methyl-3-((prop-2-yn-1-yloxy)methyl)-3,4-dihydro-2H-thieno[3,4-b][1,4]dioxepine (**EP(CCH)E**)

EP(OH)E (2.65 g, 5.52 mmol, 1 eq) was dissolved in minimal amount of acetone and then added to toluene (30 mL). Propargyl bromide (2.60 mL, 3.93 g, 33.1 mmol, 6 eq), NaOH (50 wt% in H_2O, 25 mL) and $TBAHSO_4$ (680 mg, 2 mmol, 0.4 eq) were sequentially added to the toluene solution. The mixture was allowed to stir at RT under Ar for 80 h. When TLC (DCM) indicated complete consumption of starting materials and the solution was filtered through celite to remove insoluble particles. The layers were separated and the organic layer was dried over $MgSO_4$ and filtered. Thereafter silica was added and the solvent was removed under vacuum. **EP(CCH)E** was purified through column chromatography (Pentane: DCM), DCM volume fraction varies from 50% to 100%. Fractions containing the product were combined and concentrated in vacuo. The solid was then re-dissolved in minimal amount of DCM and slowly added into a stirred solution of pentane (50 mL) yielding **EP(CCH)E** as a yellow powder.

Yield: 2.0 g, 70%, yellow powder.

^{1}H NMR (500 MHz, $CDCl_3$) δ 6.26 (s, 2H), 4.36 – 4.33 (m, 4H), 4.25 – 4.22 (m, 4H), 4.19 (d, *J* = 2.4 Hz, 2H), 4.17 (d, *J* = 11.9 Hz, 2H), 3.79 (d, *J* = 11.9 Hz, 2H), 3.69 (s, 2H), 2.44 (t, *J* = 2.4 Hz, 1H), 1.03 (s, 3H).

^{13}C NMR (126 MHz, $CDCl_3$) δ δ 144.8, 141.3, 137.3, 113.4, 110.5, 98.1, 79.9, 77.0, 74.6, 72.6, 65.3, 64.8, 59.0, 43.5, 17.3.

HRMS (APCI) calcd for $[C_{24}H_{23}O_7S_3^+, M + H]^+$: 519.0600; found: 519.0593

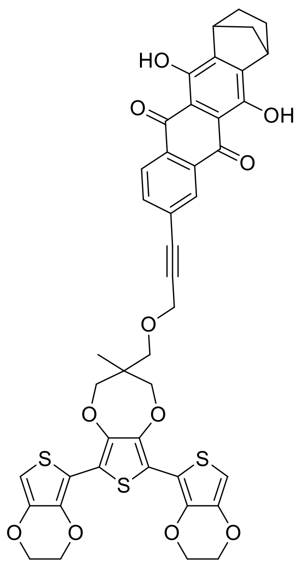

8-(3-((6,8-bis(2,3-dihydrothieno[3,4-b][1,4]dioxin-5-yl)-3-methyl-3,4-dihydro-2H-thieno[3,4-b][1,4]dioxepin-3-yl)methoxy)prop-1-yn-1-yl)-5,12-dihydroxy-1,2,3,4-tetrahydro-1,4-methanotetracene-6,11-dione (**QzH_2-EPE**)

EP(CCH)E (778 mg, 1.5 mmol, 1 eq) and **2** (706 mg, 1.5 mmol, 1 eq) were placed in a dry microwave vial (20 mL) under Ar together with $Pd(PPh_3)_2Cl_2$ (77 mg, 0.11 mmol, 0.075 eq), CuI (21 mg, 0.11 mmol, 0.075 eq) and PPh_3 (29 mg, 0.3 mmol, 0.2 eq). To these solids DIPEA (0.55 mL, 3.0 mmol, 2 eq) and DMF (8 mL) was added. The vial was caped and heated at 120 °C for 45 min. After cooling 50 mL EtOAc was added to the vial. The solution was filtered through celite to remove insoluble precipitates. The organic layer was washed with NH_4Cl (sat, 2 x 50 mL) and brine (50 mL). The organics were dried over $MgSO_4$, filtered and mixed with silica. Thereafter the solvent was removed under vacuum. The Sonogashira product was separated from the starting materials by column chromatography (Pentane: DCM), DCM volume fraction varies from 10% to 100%. Fractions containing the product were combined

and concentrated in vacuo giving in total 500 mg of the acetyl produced intermediate as a brown solid. The solid was dissolved in DCM (7 mL) to which *N*,*N*-diethylamine (3 mL) was added. After heating at 40 °C for 1 h no starting material remained (TLC – DCM). The organic layer was diluted with DCM (50 mL) and washed with 0.1M HCl (25 mL), $NaHCO_3$ (25 mL, sat) and brine (25 mL). $MgSO_4$ was added and the solution filtered. This was followed by removal of most of the DCM in vacuo. **QzH$_2$-EPE** was then precipitated by slow addition of the remaining DCM to a well-stirred pentane solution (150 mL). Subsequent filtration and drying under vacuum gave **QzH$_2$-EPE** as a red powder .

Yield: 450 mg (37%), bright red powder.

^{1}H NMR (500 MHz, $CDCl_3$) δ 13.09 (s, 1H), 13.03 (s, 1H), 8.34 (d, *J* = 1.6 Hz, 1H), 8.19 (d, *J* = 8.1 Hz, 1H), 7.78 (dd, *J* = 8.1, 1.6 Hz, 1H), 6.23 (s, 2H), 4.47 (s, 2H), 4.38 – 4.31 (m, 4H), 4.27 – 4.20 (m, 6H), 3.83 (s, 2H), 3.81 (m, 2H), 3.78 (d, *J* = 11.9 Hz, 2H), 2.03 (m, 2H), 1.81 (m, 1H), 1.60 (m, 1H), 1.29 – 1.25 (m, 2H), 1.05 (s, 3H).

^{13}C NMR (126 MHz, $CDCl_3$) δ 186.5, 186.4, 153.1, 148.4, 148.4, 144.8, 141.3, 137.4, 137.0, 133.7, 132.9, 130.1, 129.1, 127.0, 113.5, 112,3, 110.5, 98.1, 90.6, 85.1, 77.0, 72.6, 65.3, 64.8, 59.6, 49.2, 43.6, 40.8, 25.7, 17.2.

HRMS (APCI) calcd for [$C_{43}H_{35}O_{11}S_3^+$, M + H]$^+$: = 823.1336; found: 823.1304

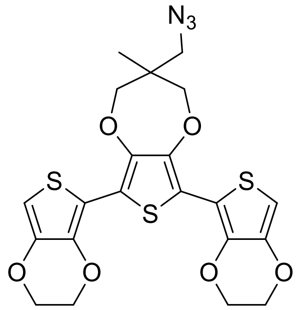

3-(azidomethyl)-6,8-bis(2,3-dihydrothieno[3,4-b][1,4]dioxin-5-yl)-3-methyl-3,4-dihydro-2H-thieno[3,4-b][1,4]dioxepine (**EP(N_3)E**)

EP(OMs)E (2.25 g, 4.0 mmol, 1 eq), NaN_3 (780 mg, 12 mmol, 3 eq) and NaI (900 mg, 6 mmol, 1.5 eq) was added to DMSO (75 mL) and heated at 100 °C for 36h. The reaction was followed by NMR and stopped when no more mesylate starting material could be detected. The solution was mixed with a 1:1 mixture of $NaHCO_3$ (sat) and water (200 mL). The resulting solid was filtered off on celite and washed with water. Then the solid was dissolved using DCM and concentrated until a few milliliter remained. The solution was then added to a stirred solution of pentane (150 mL) and filtered to give **EP(N_3)E** as a yellow powder.

Yield: 1.8 g (88%), yellow powder.

^{1}H NMR (500 MHz, $CDCl_3$) δ 6.27 (s, 2H), 4.37 – 4.33 (m, 4H), 4.25 – 4.22 (m, 4H), 4.14 (d, J = 12.0 Hz, 2H), 3.69 (t, J = 6.0 Hz, 4H), 0.96 (s, 3H).

^{13}C NMR (126 MHz, $CDCl_3$) δ 144.5, 141.4, 137.5, 113.8, 110.3, 98.3, 76.97, 65.3, 64.8, 55.2, 43.6, 17.4.

HRMS (ESI) calcd for $[C_{21}H_{20}N_3O_6S_3^+, M + H]^+$: 506.0509; found: 506.0510

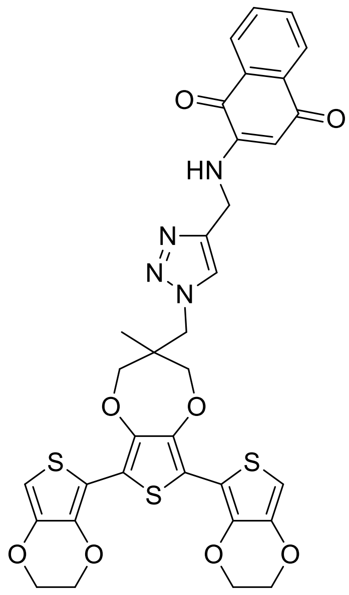

2-(((1-((6,8-bis(2,3-dihydrothieno[3,4-b][1,4]dioxin-5-yl)-3-methyl-3,4-dihydro-2H-thieno[3,4-b][1,4]dioxepin-3-yl)methyl)-1H-1,2,3-triazol-4-yl)methyl)amino)naphthalene-1,4-dione (**NQ-EPE**)

EP(N_3)E (253 mg, 0.5 mmol, 1 eq), 2-(prop-2-yn-1-ylamino)naphthalene-1,4-dion (**3**) (105 mg, 0.5 mmol, 1 eq) and CuI (20 mg, 0.1 mmol, 0.2 eq) were placed in a dry microwave vial (10 mL) under Ar. Degassed DMF (5 mL) was added and the vial was capped. The solution was heated in a microwave reactor at 100 °C for 1 h. The solution was diluted with DCM (50 mL) and washed with NH_4Cl (2 x 25 mL, sat) and brine (25 mL). The organics were dried over $MgSO_4$, filtered and mixed with silica. Purification of **NQ-EPE** was performed via column chromatography (Pentane: DCM: MeOH), DCM volume fraction (from 50% to 100%) - note the DCM contained 1% MeOH. Fractions containing the product were combined and concentrated in vacuo. The solid was dissolved in DCM (7 mL) and precipitated by slow addition to a stirred solution of pentane (150 mL). The orange/brown powder was filtered and dried under vacuum to give **NQ-EPE**.

Yield: 255 mg (71%), orange/brown powder.

^{1}H NMR (500 MHz, $CDCl_3$) δ 8.10 (dd, *J* = 7.7, 0.8 Hz, 1H), 8.06 (dd, *J* = 7.7, 0.8 Hz, 1H), 7.73 (dd, *J* = 7.6, 1.2 Hz, 1H), 7.64 (dd, *J* = 7.6, 1.2 Hz, 1H), 6.38 (m, 1H), 6.23 (s, 2H), 5.86 (s, 1H), 4.80 (s, 2H),

4.57 (d, J = 5.7 Hz, 2H), 4.37 – 4.32 (m, 4H), 4.25 – 4.21 (m, 4H), 3.99 (d, J = 12.2 Hz, 2H), 3.64 (d, J = 12.2 Hz, 2H), 0.92 (s, 3H).

^{13}C NMR (126 MHz, $CDCl_3$) δ 183.1, 181.7, 147.7, 144.1, 142.8, 141.4, 137.7, 134.9, 133.6, 132.3, 130.7, 126.5, 126.4, 124.2, 114.6, 110.0, 101.9, 98.6, 65.3, 64.7, 53.0, 43.9, 38.6, 17.3.

HRMS (ES+ TOF) calcd for [$C_{21}H_{20}N_3O_6S_3^+$ M + H]$^+$: 717.1142; found: 717.1121

S1: NMR Spectra

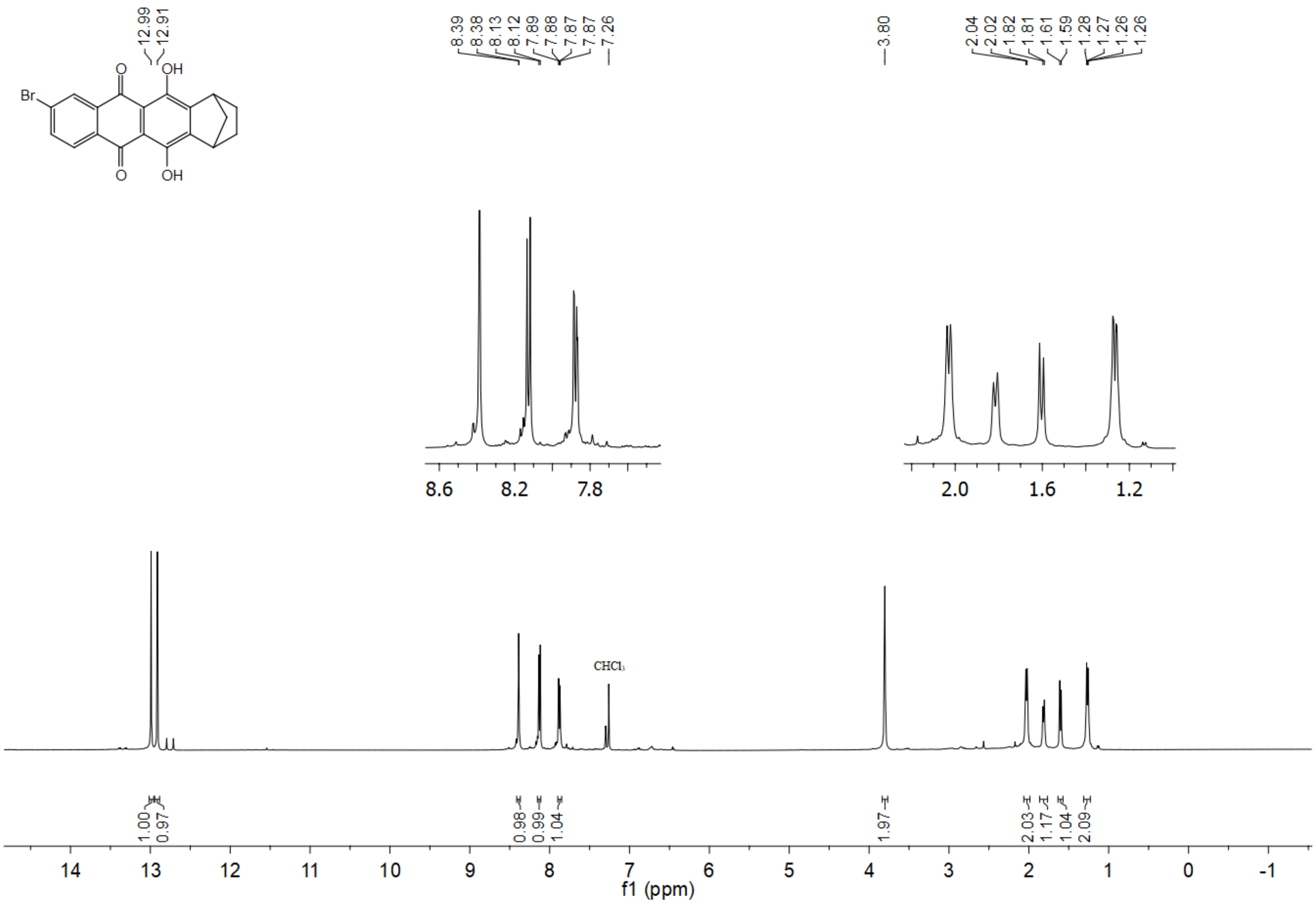

Figure S2. ^{1}H NMR spectrum (500 MHz, $CDCl_3$, RT) of 1.

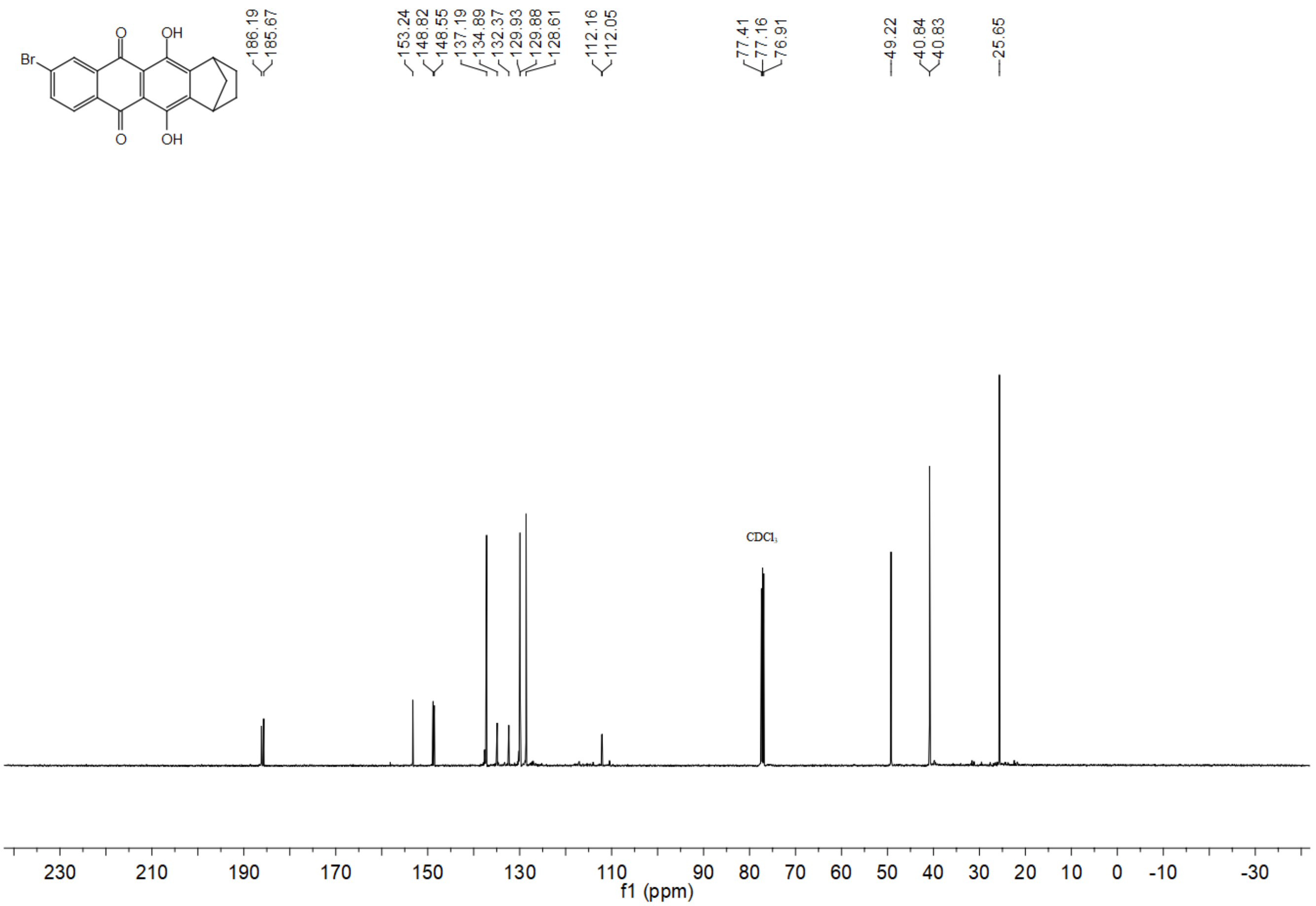

Figure S3. ^{13}C NMR spectrum (126 MHz, $CDCl_3$, RT) of **1**.

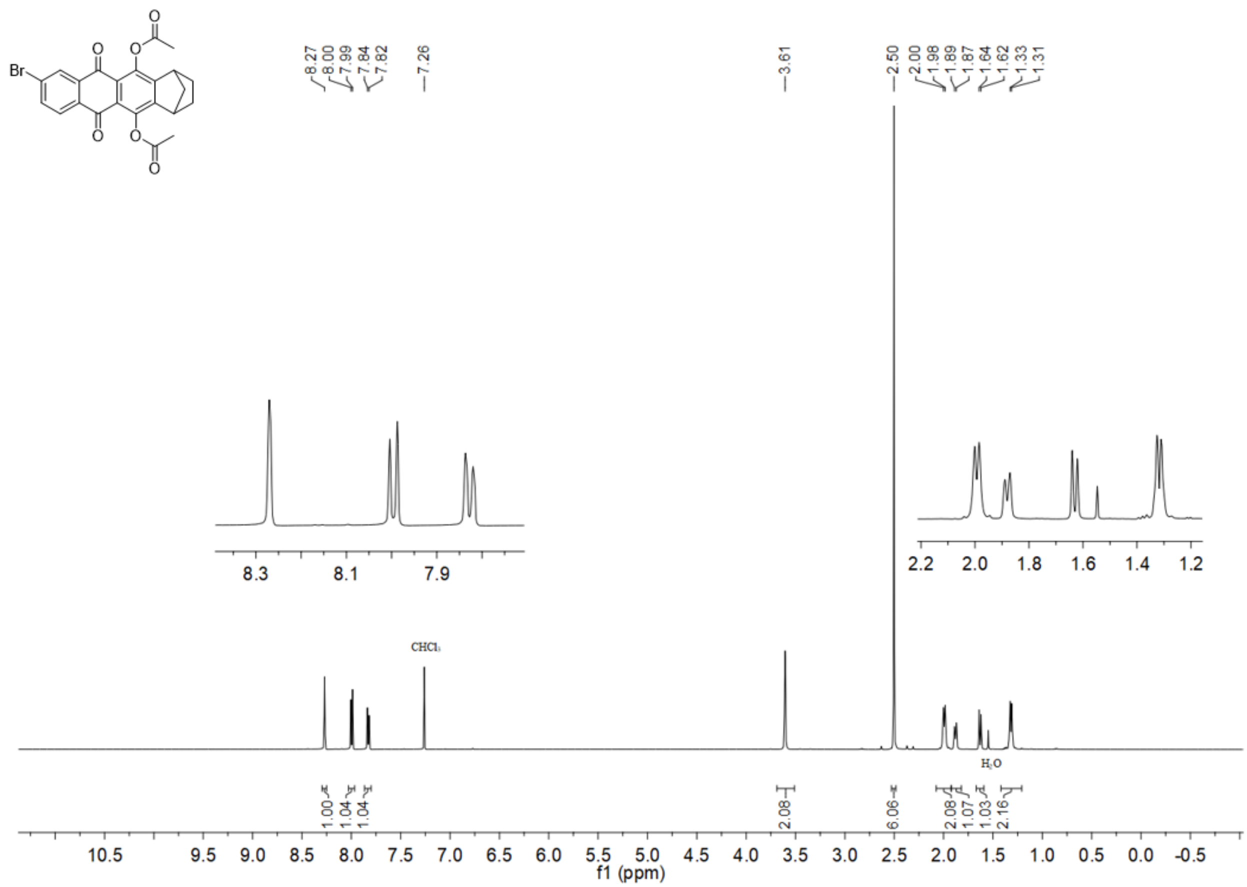

Figure S4. ^{1}H NMR spectrum (500 MHz, $CDCl_3$, RT) of **2**.

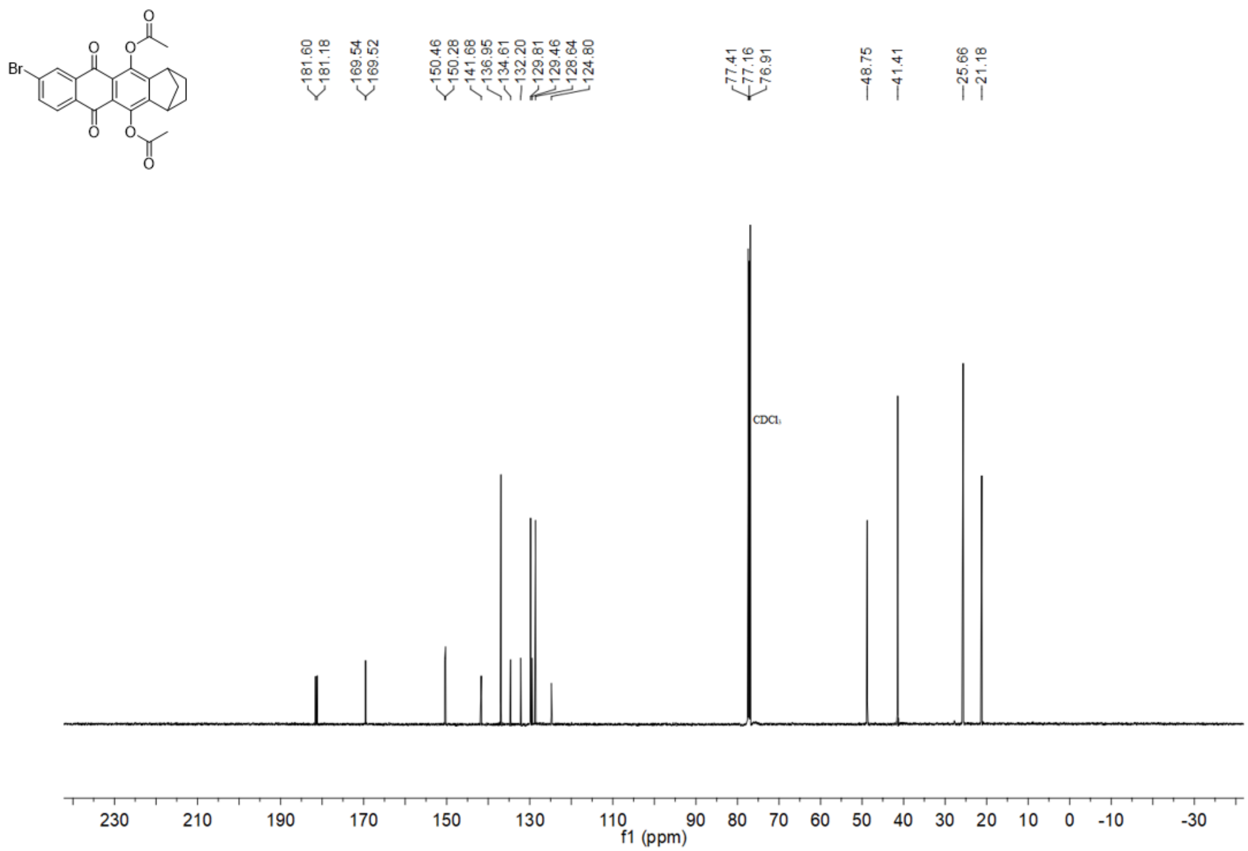

Figure S5. ^{13}C NMR spectrum (126 MHz, $CDCl_3$, RT) of **2**.

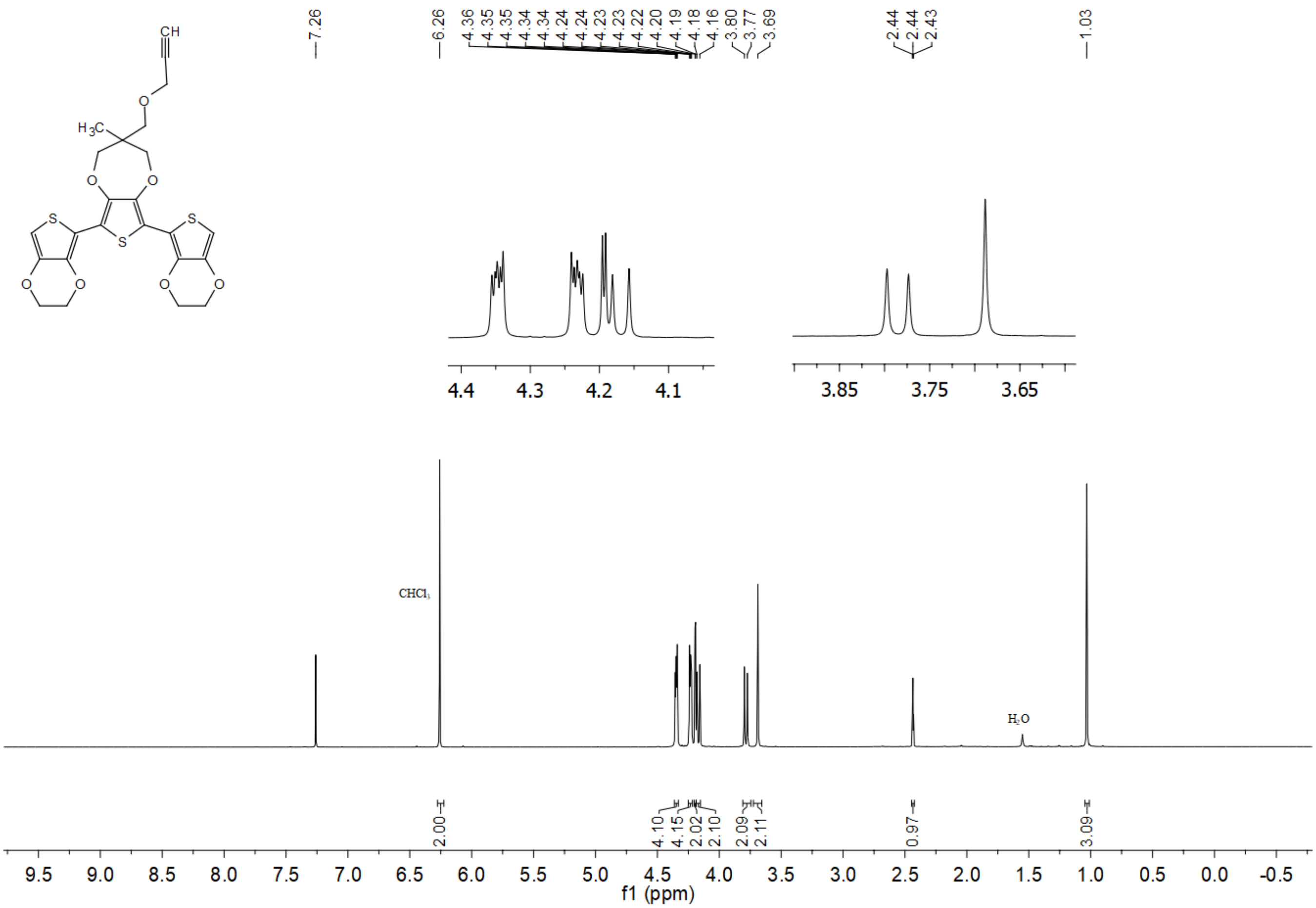

Figure S6. ^{1}H NMR spectrum (500 MHz, $CDCl_3$, RT) of **EP(CCH)E**.

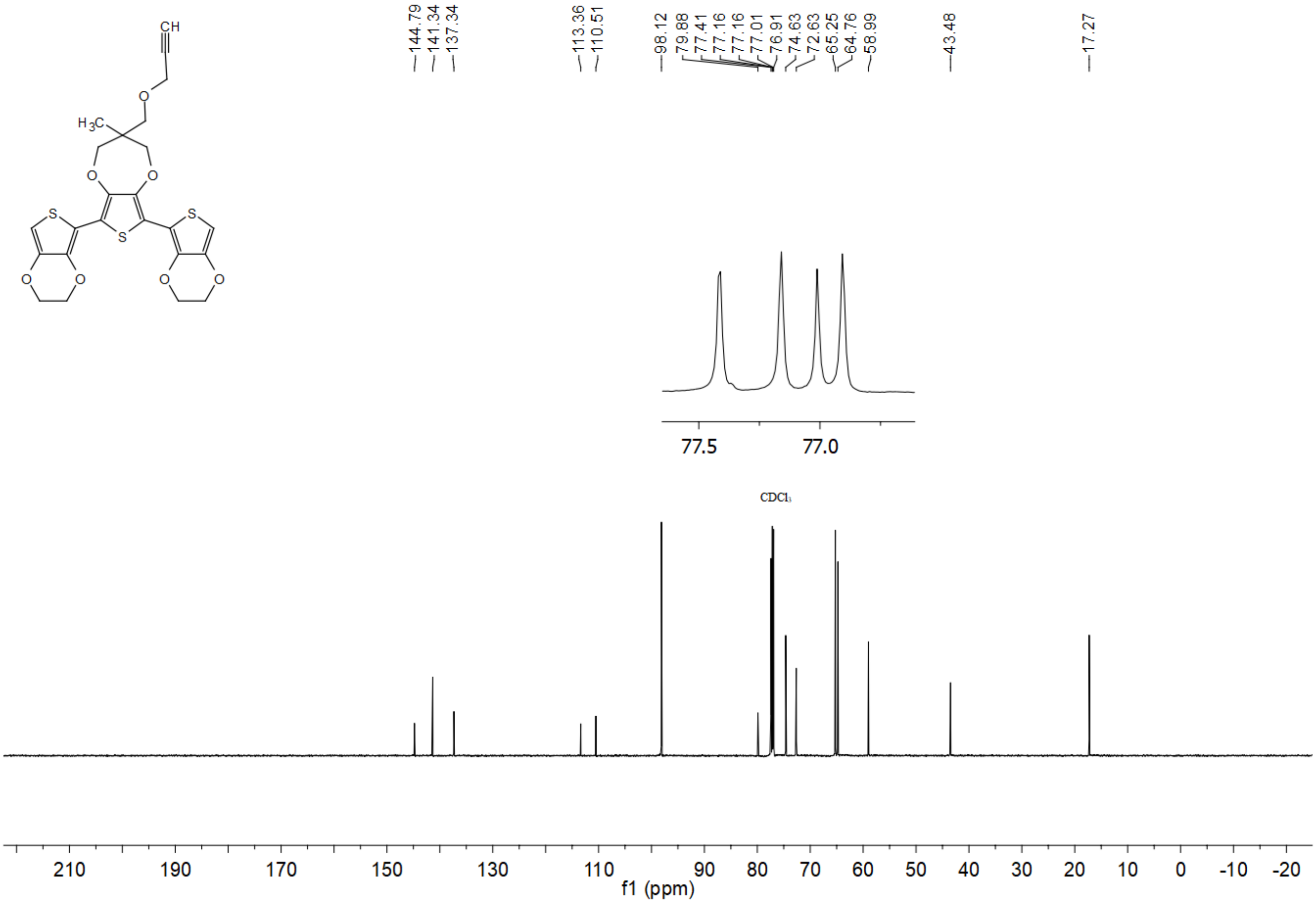

Figure S7. ^{13}C NMR spectrum (126 MHz, $CDCl_3$, RT) of **EP(CCH)E**.

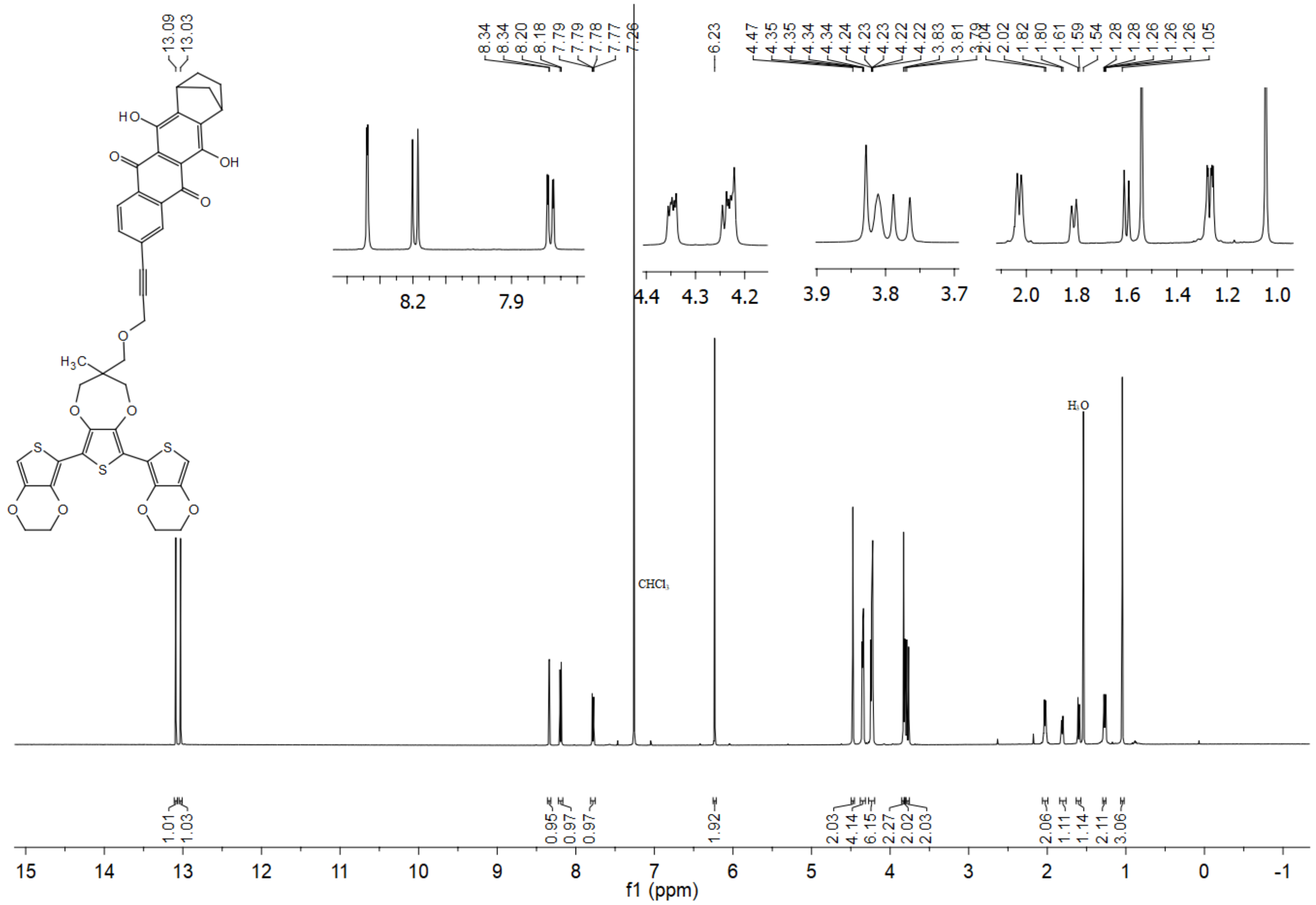

Figure S8. ^{1}H NMR spectrum (500 MHz, $CDCl_3$, RT) of **QzH2-EPE**.

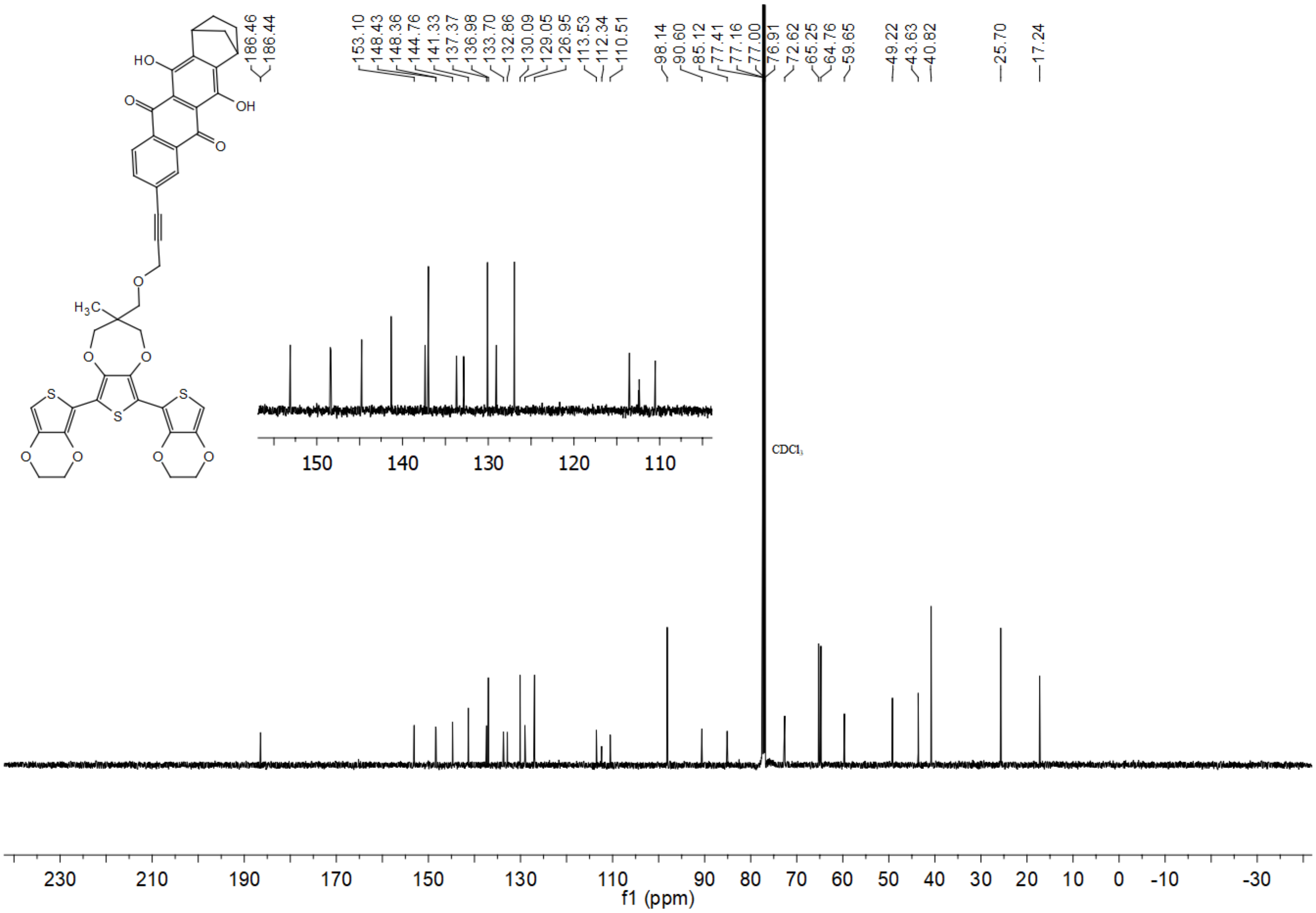

Figure S9. ^{13}C NMR spectrum (126 MHz, $CDCl_3$, RT) of **QzH$_2$-EPE**.

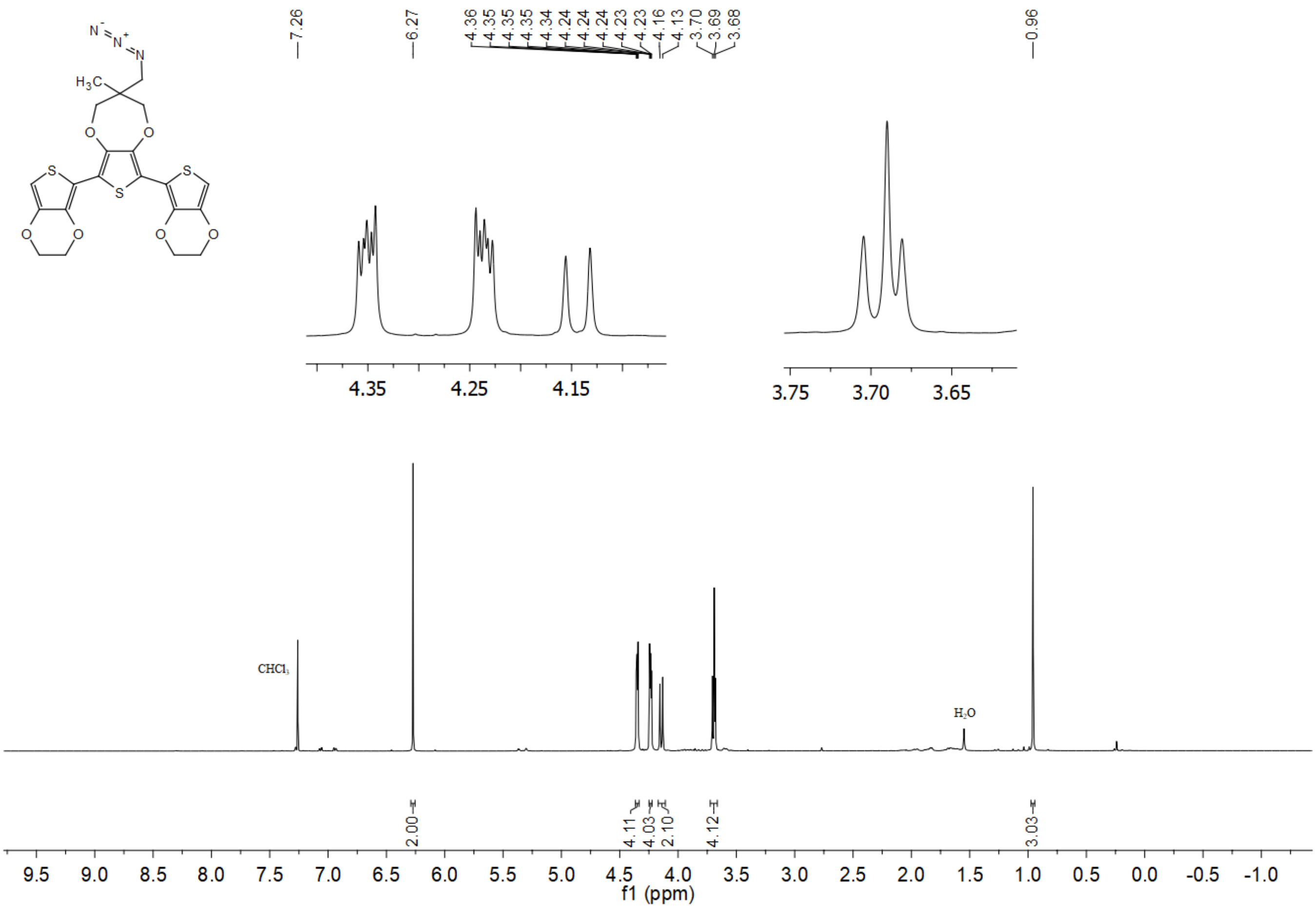

Figure S10. ^{1}H NMR spectrum (500 MHz, $CDCl_3$, RT) of **EP(N_3)E**.

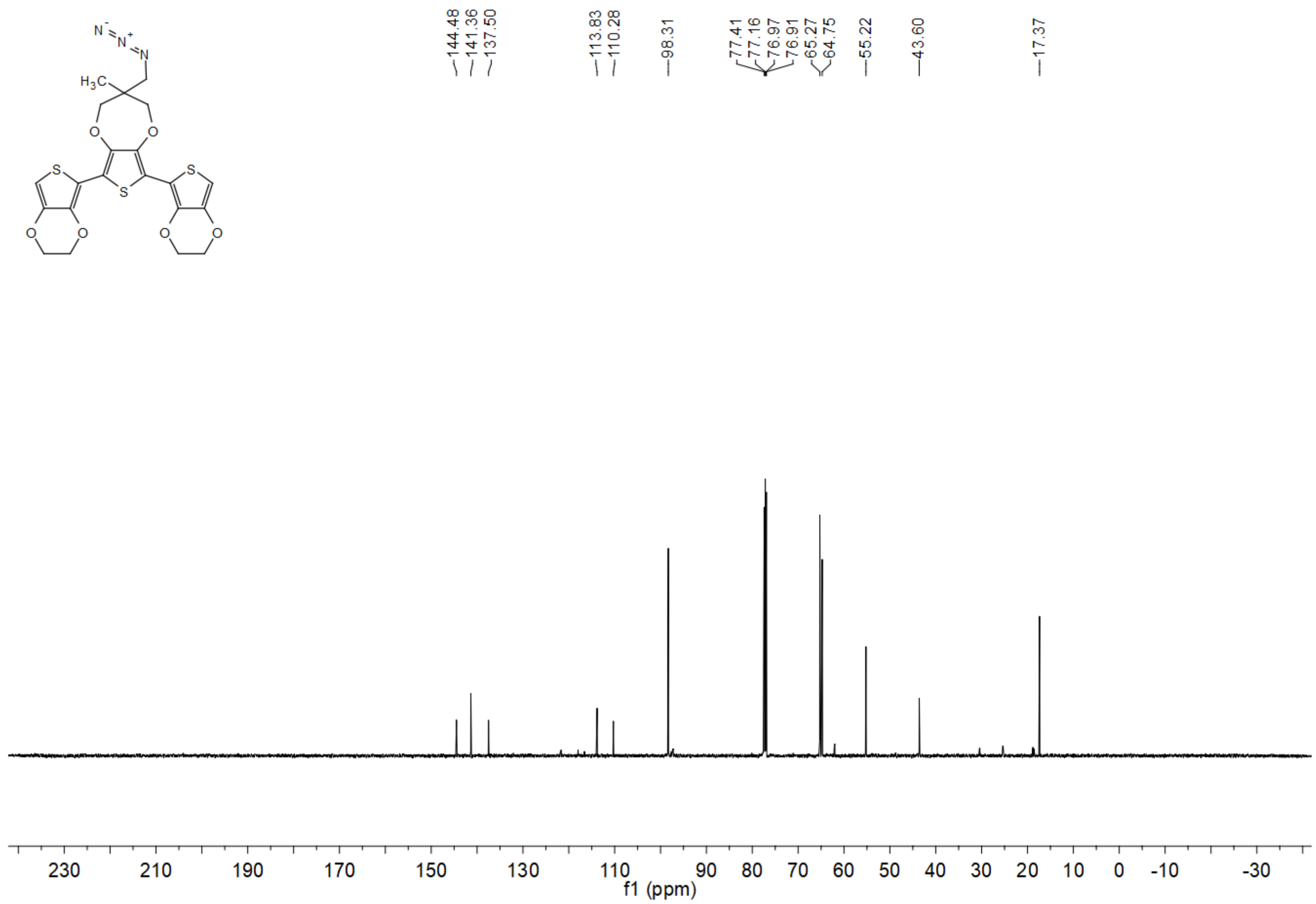

Figure S11. ^{13}C NMR spectrum (126 MHz, $CDCl_3$, RT) of **EP(N_3)E**.

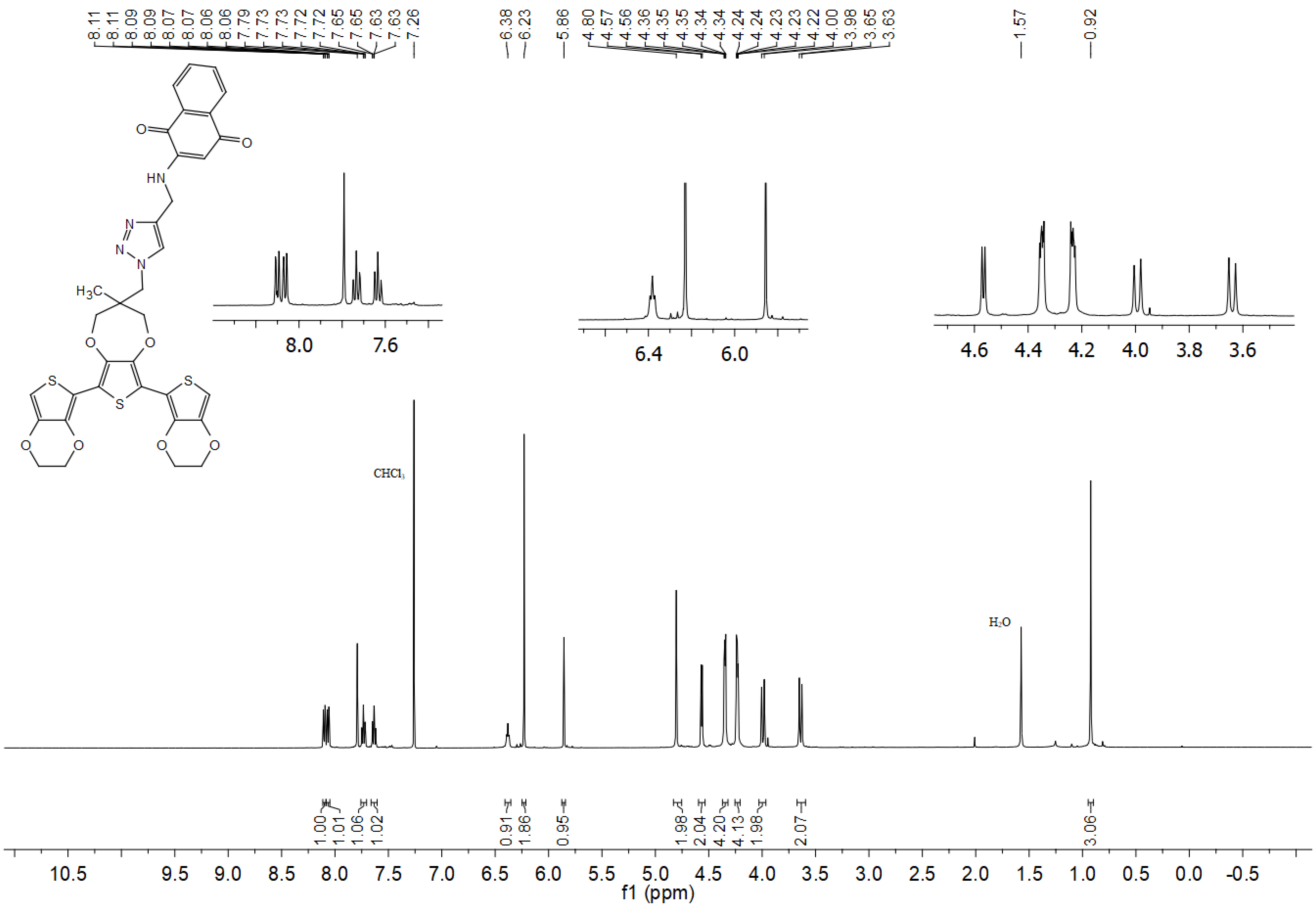

Figure S12. ^{1}H NMR spectrum (500 MHz, $CDCl_3$, RT) of **NQ-EPE**.

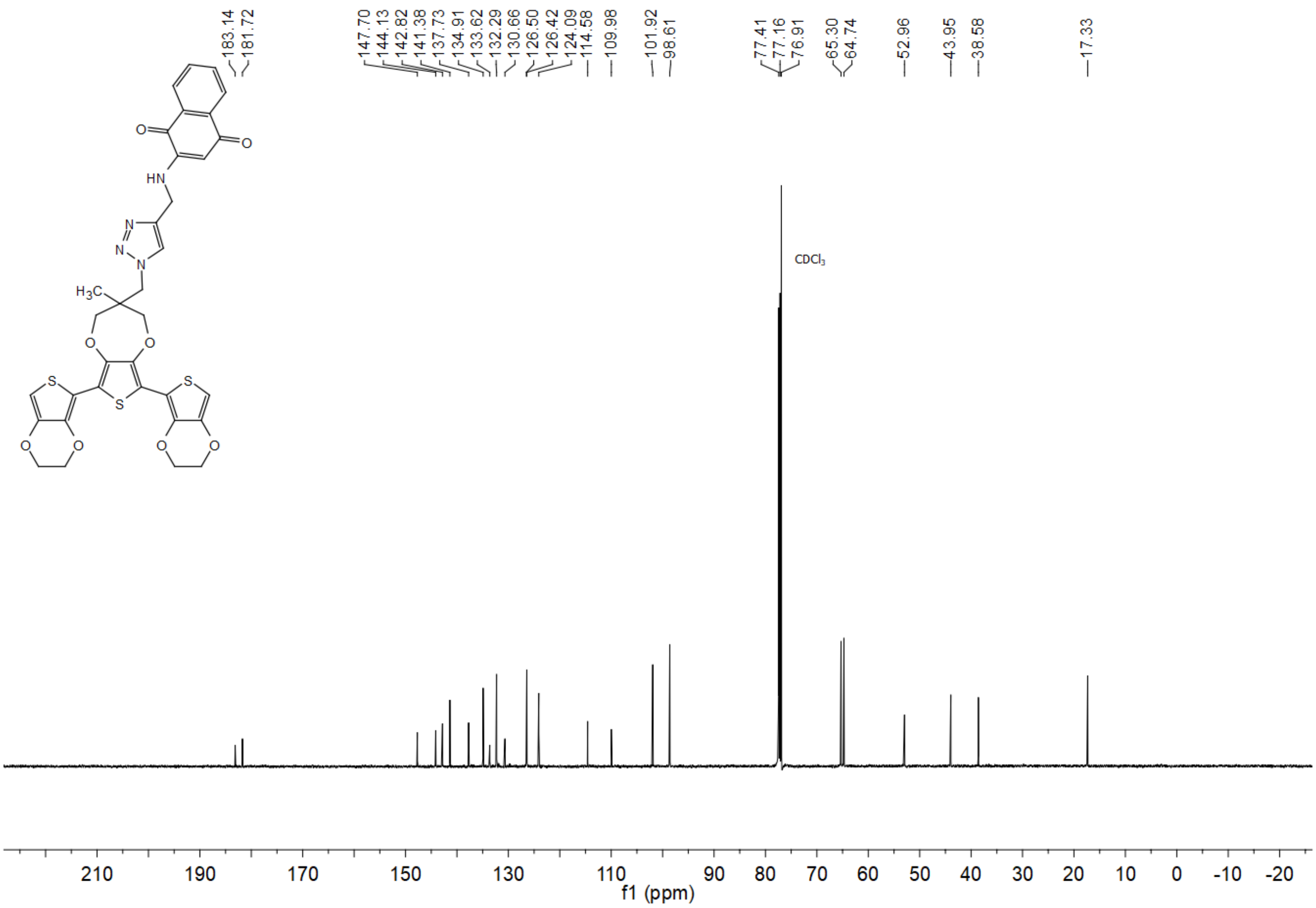

Figure S13. ^{13}C NMR spectrum (126 MHz, $CDCl_3$, RT) of **NQ-EPE**.

Section 2: Experimental procedures

All solvents and chemicals were purchased from Sigma Aldrich and were used without further purification. The MeTri-based ionic liquid electrolyte was prepared through the protonation of MeTri by bis(trifluoromethylsulfonyl)imide (HTFSI) in a glove box as described in a previous study.[3] Herein 30% degree of protonation was used due to the high conductivity ($2 \cdot 10^{-2}$ S cm^{-1} at 25 °C). In the resulting MeTriHTFSI ionic liquid electrolyte, 30% of the MeTri moieties are protonated and act as proton donors ($MeTriH^+$) while 70% are in the initial, deprotonated state (MeTri) and serve as proton acceptors. The structure of $MeTriH^+$ and MeTri are shown in Scheme 1. The TFSI anion, on the other hand, acts as charge-compensating ion for the positively charged polymer backbone during the doping process that renders the polymer conducting.

S2: Electrode preparation

QzH_2-EPE and NQ-EPE trimers were dissolved in N-methyl-2-pyrrolidone (NMP) solvent which contains 10 mg/mL polyvinylidene fluoride (PVDF), to make a 100 mg/mL trimer solution. The mass-ratio of trimer and PVDF binder was set to be 10:1. The trimer solution was drop-cast onto a glassy carbon plate (HTW, Germany, 40 mm x 8 mm x 2 mm) that was used as current collector, followed by vacuum drying at 25°C for 10 minutes to remove the solvent. The resulting glassy carbon plate was then immersed into 0.1M MeTriHTFSI/MeCN/H_2O solution.

Cyclic voltammetry (CV) was then used to electro-polymerize the trimer layer in a three-electrode setup using an Autolab PGSTAT302N potentiostat (Ecochemie, The Netherlands). The CV polymerization was conducted at a scan rate of 20 mV/s between -0.1 and 0.6 V for 6 scans in 0.1M MeTriHTFSI/MeCN/H_2O. A series of solvent mixtures with different MeCN volume fraction, 0%, 25%, 33%, 50%, 67%, 75% and 100%, were used. In 0.1M MeTriHTFSI/MeCN/H_2O solution NQ-EPE starts to dissolve when the MeCN volume fraction is higher than 67% while QzH_2-EPE starts to dissolve when

the MeCN volume fraction is higher than 75%. The glassy carbon working electrode was connected to the outer circuit as shown in Figure S14. A Pt wire was used as a counter electrode and the reference electrode consisted of an Ag wire immersed into a MeCN solution containing 0.1M MeTriHTFSI and 0.01 M $AgNO_3$, which was kept in a separate compartment with a porous glass frit. The reference electrode was calibrated against ferrocene after each set of experiments, and all potentials are reported against the ferrocene formal potential ($Fc^{+/0}$). All polymerizations were conducted in N_2 purged solutions and a N_2 pressure over the electrolyte was sustained throughout the experiment. After polymerization, the polymer-covered glassy carbon electrode was taken out from the electrolyte and was allowed to dry at ambient conditions for 30 minutes. Thick polymer films suffered from cracking after solvent evaporation, limiting the maximum mass loading to 2 mg/cm^2.

S2: Electrochemical characterization

For general electrochemical characterizations, a low polymer mass loading was used. 1.0 μL of the above 100 mg/mL trimer solution, corresponding to 0.1 mg dissolved trimer was drop-cast onto glassy carbon with the area of around 0.1 cm^2, corresponding to 1 mg/cm^2. After PDP the resulting polymer was characterized in a three-electrode setup in MeTriHTFSI electrolyte using an Autolab PGSTAT302N potentiostat (Ecochemie, The Netherlands) in a N_2-filled glovebox. The reference electrode consisted of an Ag wire immersed in 0.01 M $AgNO_3$/MeTriHTFSI solution in a separated compartment and a Pt-wire was used as counter electrode.

For *in situ* characterizations, a 10 mg/mL trimer solution excluding PVDF was used. For *in situ* conductance measurement, the working electrode was an interdigitated array (IDA) electrode with 90 pairs of gold bands on a glass substrate (10 μm between bands, 150 nm high, MicruX Technologies, Spain). *In situ* conductance measurements were conducted using an Autolab PGSTAT302N potentiostat (Ecochemie, The Netherlands) equipped with a bipotentiostat module using CV. A potential bias of 1

mV was applied between the two working electrodes.[4] The polymer conductance was investigated in 0.1M MeTriHTFSI/MeCN electrolyte. N_2 was used to purge the electrolyte solution and a N_2 pressure over the electrolyte was sustained throughout the experiment. For *in situ* electrochemical Quartz Crystal Microbalance (EQCM) measurements, the working electrode was gold coated AT-cut quartz EQCM-crystals (8.95 MHz-30 kHz, Ø 5 mm). The PDP was conducted using CV on a VersaSTAT potentiostat (VersaSTAT 4, AMETEK) while the frequency change of the EQCM crystal during CV measurement was recorded. The frequency change was subsequently converted to a mass change using the Sauerbrey equation.[5]

S2: Battery evaluation

For battery evaluations, 5.0 μL of the above 100 mg/mL trimer solution with 10 mg/mL PVDF, corresponding to 0.5 mg dissolved trimer was drop-cast onto a glassy carbon yielding a covered area of 0.25-0.35 cm^2, with a corresponding mass loading of 1.4-2.0 mg/cm^2. The thickness of the prepared polymer films are around 0.3 mm. The prepared poly (QzH_2-EPE) and poly (NQ-EPE) electrodes were assembled into a pouch cell in a N_2-filled glove box. A glass microfiber filter (CAT NO. 1820-055, Whatman) was used as separator and MeTriHTFSI was used as electrolyte. In order to evaluate the properties of the two polymers separately, two types of cells were prepared; one was capacity-limited by the poly (NQ-EPE) anode and the other one by the poly (QzH_2-EPE) cathode. The mass-ratio of active materials at the two electrodes was 1:4. In the anode-limiting case the total theoretical capacity of cathode side is 3.5 times of the anode while in the cathode-limiting case the total theoretical capacity of anode side is 4.6 times of the cathode. The electrodes were pre-discharged by CV before they were assembled into a battery. The battery performance was investigated using an Autolab PGSTAT302N potentiostat.

S2: Other characterization

Scanning Electron Microscopy (SEM) was carried out using a Leo Gemini 1550 FEG SEM instrument (Zeiss, Germany) equipped with both an in-lens and EDX detector. The sample was pre-coated with a conductive gold layer prior to imaging. FTIR spectroscopy absorption of trimer powder and ex-situ polymer was recorded on a BRUKER Tensor 27 with a Bruker platinum *ex situ* ATR mode, 64 background scans were recorded prior to the measurement and subtracted from the sample spectra composed of 64 scans.

For *in situ* FTIR measurements, a conductive gold layer sputtered onto ZnSe crystal (45º electrode) was used as working electrode. The polymer potential is set by chronoamperometry in 0.1M MeTriHTFSI/MeCN on a CHI instrument (660D potentiostat Ireland). *In situ* FTIR spectroscopy absorption was performed on a Bruker Tensor 27 FTIR equipped with VeeMAX III. 64 background scans were recorded prior to the measurement and subtracted from the sample spectra composed of 64 scans.

Section 3: Post-deposition polymerization (PDP)

Figure S14 shows electro-polymerization setup of trimer layer. The glassy carbon with trimer layer was put into 0.1M MeTriHTFSI/MeCN/H_2O (Vol MeCN: 67% for NQ-EPE, 75% for QzH_2-EPE). solution where the trimer layer does not dissolve. The trimer layer was electro-polymerized by cyclic voltammetry in the three-electrode setup.

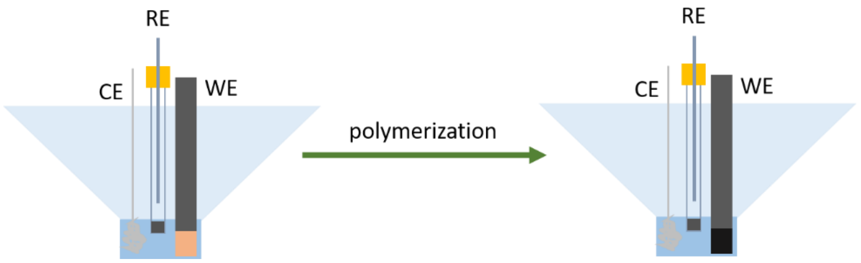

Figure S14. Scheme of the electro-polymerization setup in a three-electrode setup.

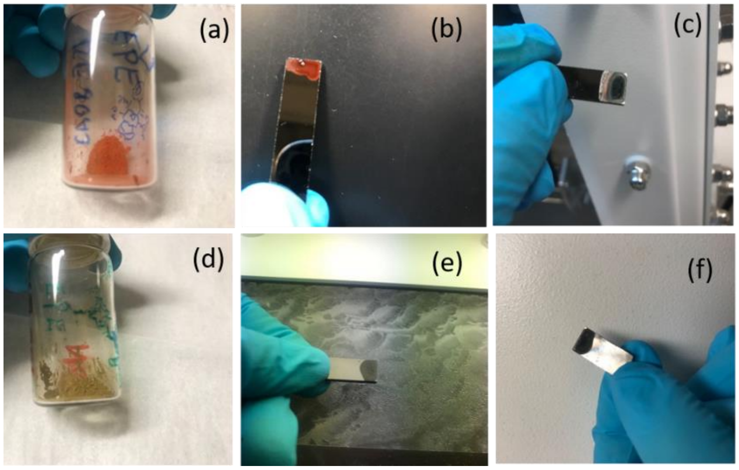

Figure S15. Photograph of (a) QzH_2-EPE trimer powder, (b) QzH_2-EPE trimer film, (c) poly (QzH_2-EPE) film, (d) NQ-EPE trimer powder, (e) NQ-EPE trimer film, (f) poly (NQ-EPE) film.

S3: Cyclic voltammogram

Polymerization cyclic voltammograms (CVs) of QzH_2-EPE shows that current rises from 0.2 V in the first anodic scan (Figure S16), which is attributed to the oxidation of neutral state trimer. The rectangular-shape current between 0 V and 0.2 V is attributed to the doping of resulting polymer, which builds up with polymerization. The cathodic peak centered at around 0.15 V is attributed to the Qz/QzH_2 reduction, which also builds up with the polymerization. The anodic peak of Qz/QzH_2 redox transfer is at around 0.6 V. The polymerization was completed in 6 scans.

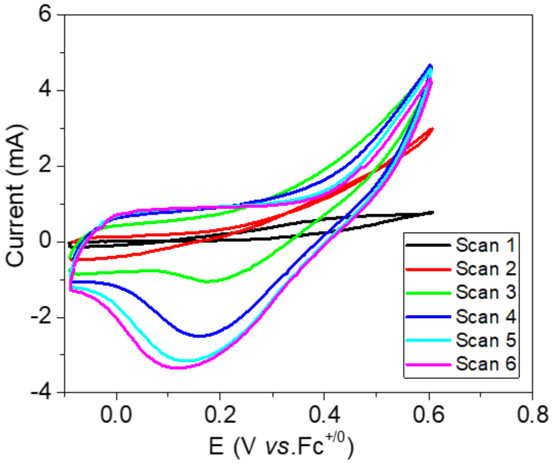

Figure S16. Polymerization cyclic voltammograms of 0.5 mg QzH_2-EPE film on glassy carbon at 20 mV/s in 0.1M MeTriHTFSI/MeCN/H_2O (Vol MeCN: 75%).

S3: Solvent-uptake in *in situ* EQCM measurement

Unlike traditional polymerization from a soluble monomer solution, the PDP method is to polymerize an insoluble trimer film. The mass increase upon polymerization results from the solvent or charge-compensating counter ion uptake, where the solvent-uptake is the dominant mass-transfer process in the initial stage of polymerization and can be roughly estimated. Cyclic voltammograms show a big anodic peak centered at 0.2 V (Figure S17), which is attributed to the oxidation of QzH_2-EPE. Above 0.2 V mass increases extensively in response to the polymerization. Above 0.4 V the speed of mass increase slows down as a result of the proton release from Qz/QzH_2 oxidation. The proton release amount in the anodic scan can be reversibly uptaken in the following cathodic scan as Qz is reversibly reduced to QzH_2. The polymer doping induced TFSI anion uptake in the anodic scan can also be reversibly expulsed in the following cathodic scan as the positively charged polymer backbone is reduced to neutral state. The net mass change between the anodic scan and cathodic scan is thus the amount of solvent uptaken, which is 4.9 µg, corresponding to 49 wt% of the trimer mass.

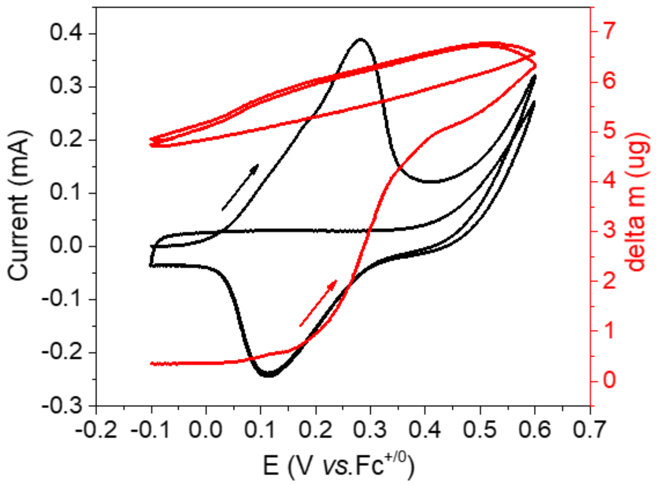

Figure S17. Cyclic voltammograms (black) and corresponding mass change (red) during the polymerization of 10 μg QzH_2-EPE on EQCM Au-electrode at a scan rate of 20 mV/s in 0.1M MeTriHTFSI/MeCN/H_2O (Vol MeCN: 75%). The arrow indicates the anodic scan.

S3: Conductance builds up in *in situ* conductance measurement

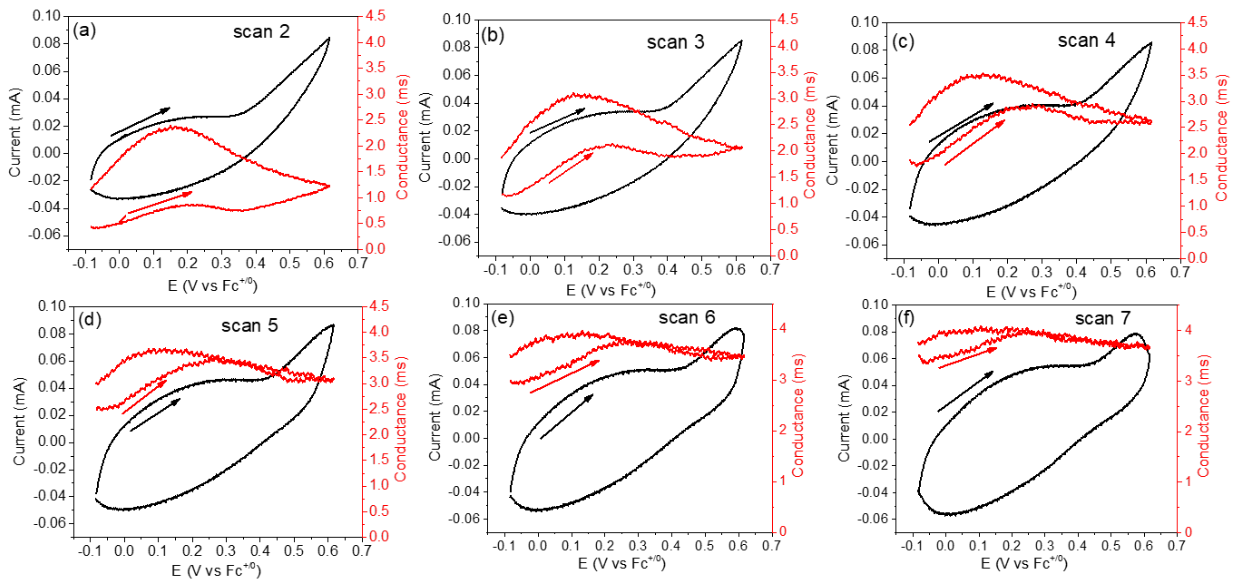

Figure S18. (a) 2nd, (b) 3rd, (c) 4st, (d) 5st, (e) 6st, (f) 7st polymerization scan during polymerization of 10 μg NQ-EPE on IDA Au-electrode at a scan rate of 50 mV/s in 0.1M MeTriHTFSI/MeCN/H_2O (Vol MeCN: 67%). The arrow indicates the anodic scan.

In order to monitor the conductance evolution during polymerization, we post-deposited the trimers onto interdigitated array (IDA) electrode composed of two interdigitated electrodes separated by 10 μm. The IDA electrode with QzH_2-EPE trimer was vacuum dried and transferred to 0.1M MeTriHTFSI/MeCN/H_2O (Vol MeCN: 75%), where the QzH_2-EPE trimer layer does not dissolve. The trimer layer was polymerized into polymer using cyclic voltammetry method. 1 mV voltage bias was applied between the two working electrodes, the current flow on the resulting polymer can be monitored as a function of applied potential. The conductance of polymer can be calculated according to our previous report[6]. Figure S19 shows the conductance response during the polymerization of QzH_2-EPE.

In the first anodic scan, the polymerization current rises from 0.3 V, the conductance rises as a result of the trimer polymerization. In the following cathodic scan, conductance keeps increasing above 0.1 V as a result of continuous polymerization. The polymerization was completed after 4 scans, however, the phenomena that over-doping-induced conductance decrease at a certain potential still exists.

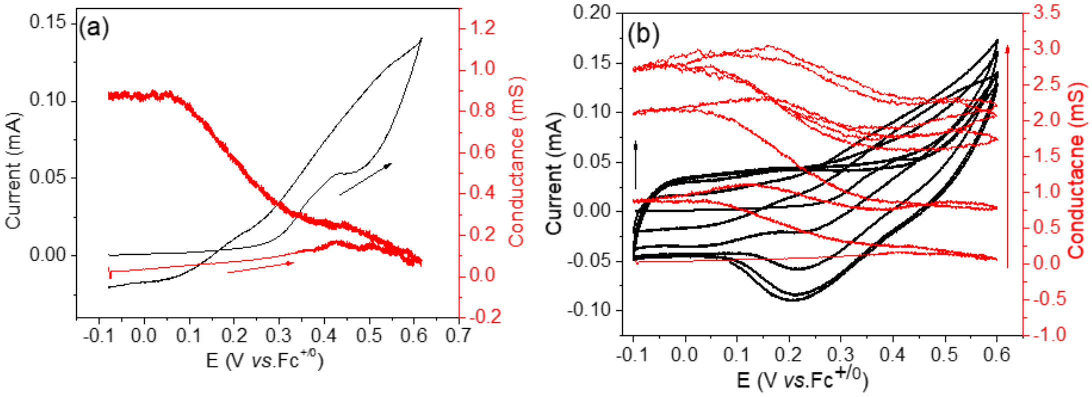

Figure S19. Conductance (red) and cyclic voltammograms response (black) during (a) the first polymerization scan, (b) first five polymerization scans of QzH_2-EPE on IDA Au-electrode at a scan rate of 50 mV/s in 0.1M MeTriHTFSI/MeCN/H_2O (Vol MeCN: 75%), the arrow indicates the building up of current and conductance.

Section 4: Polymerization solution optimization

S4: Polymer morphology evolution

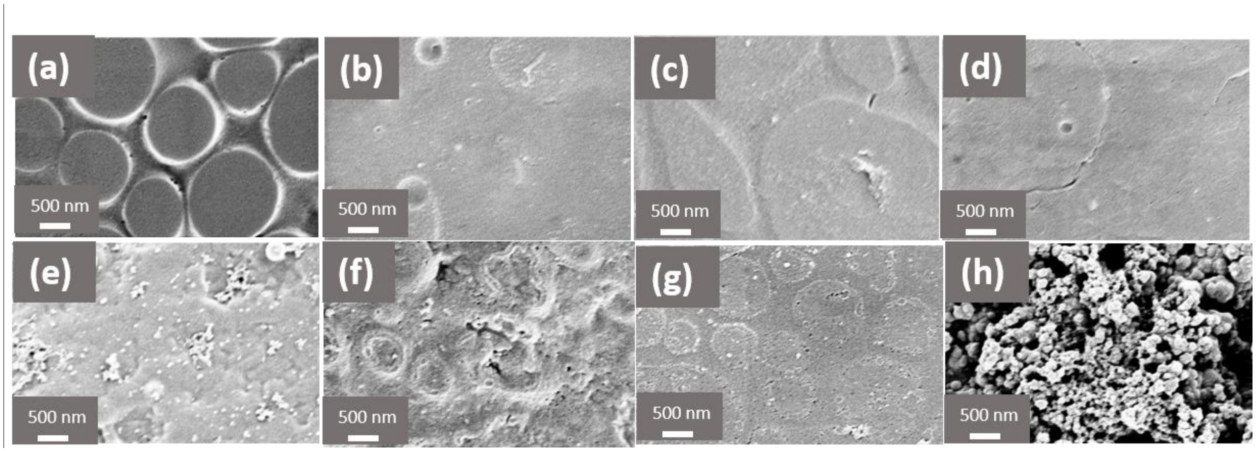

Figure S20. SEM image of QzH_2-EPE trimer (a), poly (QzH_2-EPE) polymerized in 0% (b), 25% (c), 33% (d), 50% (e), 67% (f), 75% (g), 100% (h) volume fraction of MeCN in 0.1M MeTriHTFSI/MeCN/H_2O. All the sample are tested on glassy carbon current collector.

S4: Polymer length

The polymer length can be estimated from the polymerization charge, which was recorded during polymerization. We use only 10 µg trimer and a slow scan rate of 8 mV/s to make sure that all the trimers are polymerized in the first anodic scan. In the first anodic scan, the observed oxidative charge is a sum up of polymerization charge and doping charge of the resulting polymer. By assuming that the doping charge of polymer backbone is reversibly released in the cathodic scan, we take the net charge between the anodic scan and cathodic scan as the polymerization charge. The polymerization cutoff window is 0.4 V to avoid any irreversible oxidation of electrolyte. For QzH_2-EPE polymerization, the low polymerization window can also avoid the Qz/QzH_2 oxidation, which would contribute to the overall anodic charge. The polymerization charge of 10 µg QzH_2-EPE ($1.2*10^{-8}$ mol) is estimated to be $1.62*10^{-8}$ mol (Figure S21). The polymer length can be calculated according to the following equation:

$$x = \frac{2n_t}{2n_t - n_e}$$

n_e (mol) is the sum of total electron transfer, n_t (mol) is the sum of trimer unit. Each polymer length contains x trimer unit on average, the average chain number is n_t/x. The total number of electron transfer per chain is 2*(x-1) and the total number of electron transfer is 2*(x-1)* n_t/x, which is n_e. The calculated average polymer length is 3.1 trimer per chain, corresponding to 9 EDOT unit. While the polymerization charge of 10 µg NQ-EPE (1.3*10^{-8} mol) is 2.02*10^{-8} mol (Figure S22), the calculated polymer length is 14 EDOT unit. The effect of MeCN fraction in the polymerization solution (0.1M MeTriHTFSI/MeCN/H_2O) on the resulting polymer length was also investigated and listed in Table S1 and S2.

From the relatively constant current originating from polymer doping the backbone capacitance for poly (QzH_2-EPE) can be estimated to 189 F/g. This corresponds to a doping level (per thiophene unit) of 0.54 charges per monomer per unit potential. Using a polymer onset-doping potential of -0.65 V the doping level after polymerization, i.e. at -0.1 V (vs. $Fc^{+/0}$), was estimated to 0.3 charges per thiophene unit. For poly (NQ-EPE) the capacitance was estimated to 184 F/g and the doping level after the polymerization process was estimated to 0.27 charges per thiophene unit.

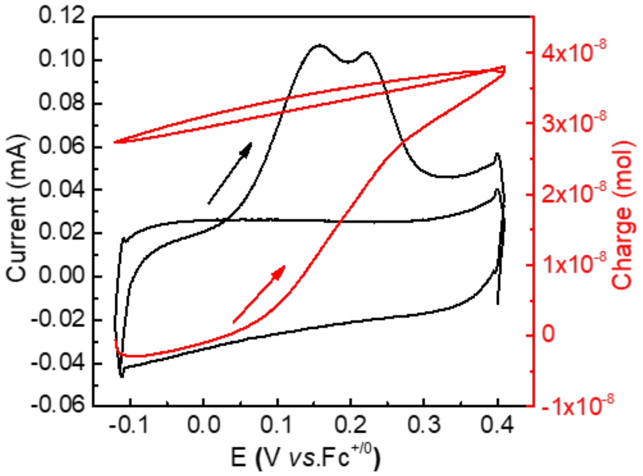

Figure S21. Cyclic voltammograms (black) and corresponding charge (red) during polymerization of 10 μg QzH_2-EPE on glassy carbon at a scan rate of 8 mV/s in 0.1M MeTriHTFSI/MeCN/H_2O (Vol MeCN: 75%), the arrow indicates the first anodic scan.

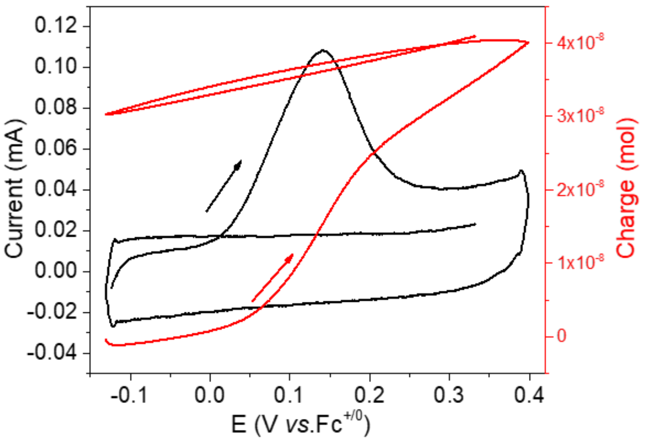

Figure S22. Cyclic voltammograms (black) and corresponding charge (red) during polymerization of 10 μg NQ-EPE on glassy carbon at a scan rate of 8 mV/s in 0.1M MeTriHTFSI/MeCN/H_2O (Vol MeCN: 67%), the arrow indicates the first anodic scan.

Table S1. Polymerization data of 10 μg NQ-EPE polymerized in 0.1M MeTriHTFSI/MeCN/H_2O with different MeCN volume fractions.

MeCN volume fraction	0%	25%	33%	50%	67%
number of trimer (mol)	$1.30*10^{-8}$	$1.30*10^{-8}$	$1.30*10^{-8}$	$1.30*10^{-8}$	$1.30*10^{-8}$
oxidative charge (mol C)	$2.95*10^{-8}$	$4.07*10^{-8}$	$3.91*10^{-8}$	$4.68*10^{-8}$	$4.09*10^{-8}$
reductive charge (mol C)	$1.74*10^{-8}$	$2.52*10^{-8}$	$2.13*10^{-8}$	$2.76*10^{-8}$	$2.07*10^{-8}$
polymerization charge (mol C)	$1.21*10^{-8}$	$1.54*10^{-8}$	$1.78*10^{-8}$	$1.92*10^{-8}$	$2.02*10^{-8}$
average polymer length (trimer)	1.9	2.5	3.2	3.8	4.5
number of EDOT unit per chain	6	8	10	11	14

Table S2. Polymerization data of 10 μg QzH_2-EPE polymerized in 0.1M MeTriHTFSI/MeCN/H_2O with different MeCN volume fractions.

MeCN volume fraction	0%	25%	33%	50%	67%	75%
number of trimer (mol)	$1.2*10^{-8}$	$1.2*10^{-8}$	$1.2*10^{-8}$	$1.2*10^{-8}$	$1.2*10^{-8}$	$1.2*10^{-8}$

oxidative charge (mol C)	$2.083*10^{-8}$	$2.79*10^{-8}$	$3.28*10^{-8}$	$3.31*10^{-8}$	$3.53*10^{-8}$	$3.88*10^{-8}$
reductive charge (mol C)	$1.68*10^{-8}$	$2.16*10^{-8}$	$2.18*10^{-8}$	$1.99*10^{-8}$	$2.07*10^{-8}$	$2.26*10^{-8}$
polymerization charge (mol C)	$4.043*10^{-8}$	$6.423*10^{-8}$	$1.11*10^{-8}$	$1.33*10^{-8}$	$1.46*10^{-8}$	$1.62*10^{-8}$
average polymer length (trimer)	1.2	1.4	1.9	2.2	2.8	3.1
number of EDOT unit per chain	4	4	6	7	8	9

Figure S23a shows the galvanostatic charge-discharge curves of poly (NQ-EPE) which was polymerized in 0.1M MeTriHTFSI/H_2O using PDP method. The electrode was firstly discharged (black), showing a slight slope with the capacity of 10 mAh/g. In the following charging process, a plateau centered at -0.3 V was observed, which is attributed to NQ/NQH_2 oxidation. Further charging to high potential exhibited a plateau at 0.1 V, which is attributed to further polymerization (oxidation) of the obtained short polymer in MeTriHTFSI ionic liquid. In the second discharge cycle NQ/NQH_2 exhibited a discharge capacity of 25 mAh/g, which increased a lot compared to the first cycle due to further polymerization. Figure S23b shows the galvanostatic charge-discharge curves of poly (NQ-EPE) which was polymerized in 0.1M MeTriHTFSI/MeCN/H_2O (Vol MeCN: 25%) using PDP method. The capacity kept increasing upon cycling, which may be attributed to the slow wetting-assisted activation of polymer in sticky MeTriHTFSI electrolyte. When the MeCN volume fraction in 0.1M MeTriHTFSI/MeCN/H_2O polymerization solution increased to 50%, the plateau capacity of NQ/NQH_2 redox transfer is close to its theoretical capacity (78 mAh/g).

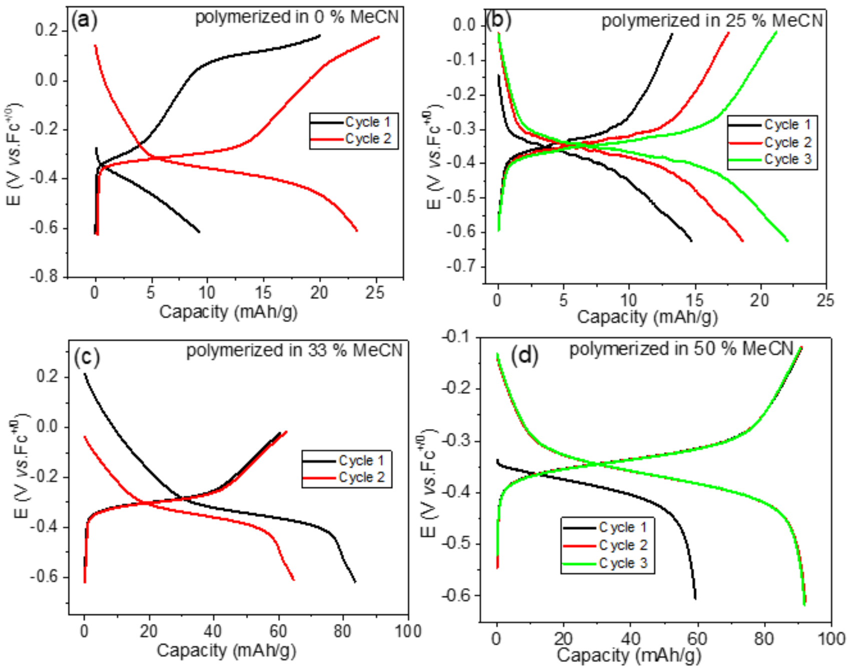

Figure S23. Galvanostatic charge-discharge curves of 0.1 mg poly (NQ-EPE) tested on glassy carbon in three-electrolyte setup in MeTriHTFSI electrolyte at a current density of 0.3A/g. Poly (NQ-EPE) was obtained from the polymerization of NQ-EPE trimer in MeTriHTFSI/MeCN/H_2O with MeCN volume fraction of (a) 0%, (b) 25%, (c) 33%, (d) 50%.

The formal potential of Qz/QzH_2 in MeTriHTFSI electrolyte is 0.45 V, which is relatively higher than the trimer oxidation potential, signifying that we cannot exclude further polymerization in MeTriHTFSI electrolyte. Figure S24a shows the galvanostatic charge-discharge curves of poly (QzH_2-EPE) which was polymerized in 0.1M MeTriHTFSI/H_2O using PDP method. Partial dissolution of polymer in MeTriHTFSI was observed as a result of short polymer length. The electrode was firstly discharged (black), showing a negligible discharge capacity without apparent discharge plateau. The following charging process exhibited a slope without defined plateau, which is attributed to the further polymerization of the obtained short poly (QzH_2-EPE). It´s worthy to note that a drastic dissolution was observed during the further polymerization process. Figure S24b shows the galvanostatic charge-

discharge curves of poly (QzH_2-EPE) which was polymerized in 0.1M MeTriHTFSI/MeCN/H_2O (Vol MeCN: 25%) using PDP method. The resulting polymer also shows further polymerization upon 0.4 V as well as visible dissolution of the obtained short oligomer. This further polymerization in MeTriHTFSI disappears when the MeCN volume fraction was above 33%, (Figure S24c)

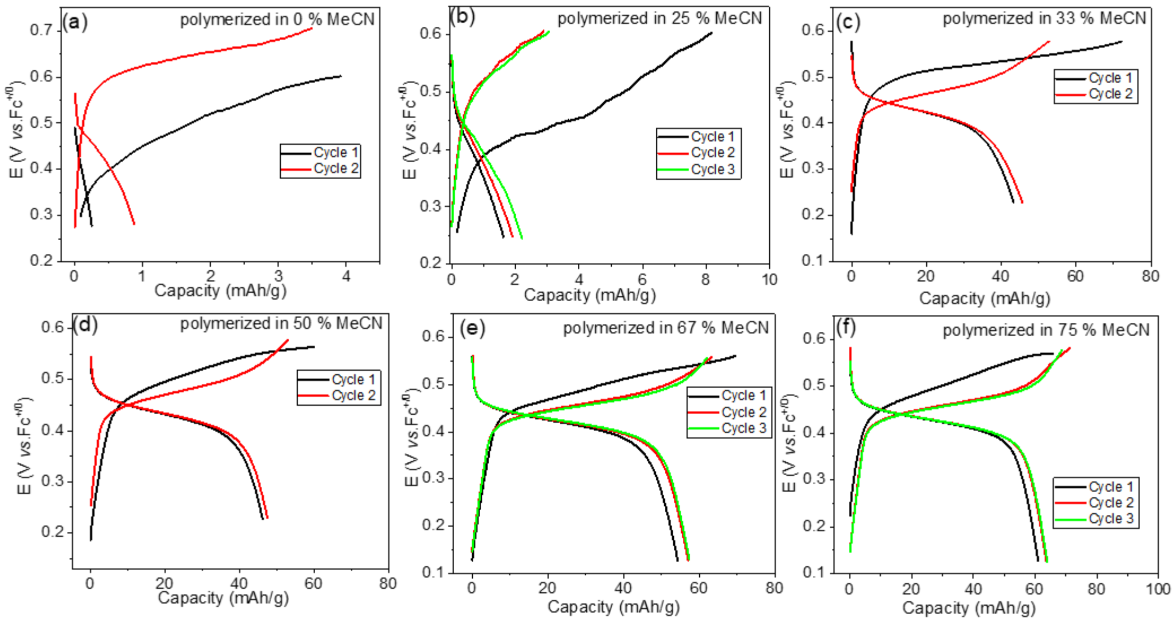

Figure S24. Galvanostatic charge-discharge curves of 0.1 mg poly (QzH_2-EPE) tested on glassy carbon in three-electrolyte setup in MeTriHTFSI electrolyte at a current density of 0.3 A/g. poly (QzH_2-EPE) was obtained from the polymerization of QzH_2-EPE in 0.1M MeTriHTFSI/MeCN/H_2O with MeCN volume fraction of (a) 0%, (b) 25%, (c) 33%, (d) 50%, (e) 67%, (f) 75%.

S4: oxidation potential of neutral state trimer

Figure S25 shows the polymerization CVs of 10 μg QzH_2-EPE in 0.1M MeTriHTFSI/MeCN/H_2O solution with different MeCN volume fractions. The polymerization was completed in the first anodic scan and only rectangular-shape polymer backbone doping/dedoping charge is observed in the second anodic scan. The redox potential of QzH_2-EPE trimer shifts negatively with the MeCN volume fraction

in the polymerization solution. The anodic peak of trimer redox reaction is at 0.45 V in 0% MeCN electrolyte and gradually shifts negatively to 0.25 V in 67% MeCN electrolyte and 0.15 V in 75% MeCN electrolyte.

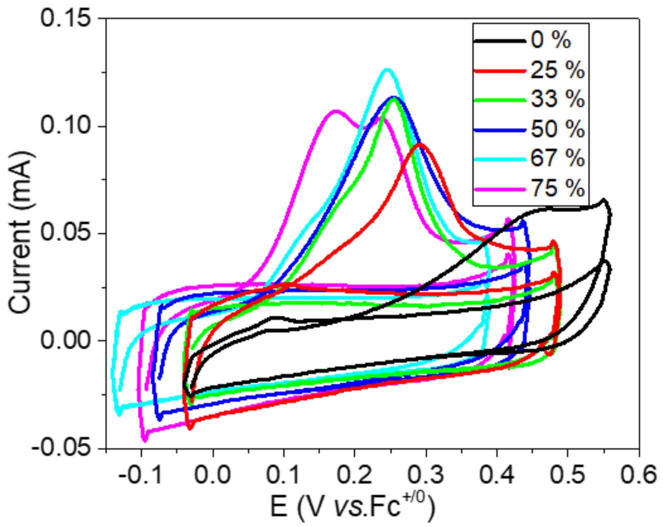

Figure S25. Polymerization cyclic voltammograms of 10 µg QzH_2-EPE on glassy carbon at 8 mV/s in 0.1M MeTriHTFSI/MeCN/H_2O with different MeCN volume fractions.

Section 5: Polymer characterization

S5: *ex situ* ATR and *in situ* FTIR

The ATR spectra of the resulting poly (QzH_2-EPE) and poly (NQ-EPE) were compared to the corresponding starting materials (QzH_2-EPE and NQ-EPE) as well as to the non-substituted trimer (EP(OH)E). Prominent peaks that appeared in the NQ-EPE or in the poly (NQ-EPE) spectra but not in EP(OH)E are marked with straight lines and their respective wavenumbers are indicated in Figure S26. These peaks assigned to pendants were preserved in the polymer albeit broadened and often red-shifted by a few wavenumbers, indicating that the structure of the pendant groups were preserved in the polymer.

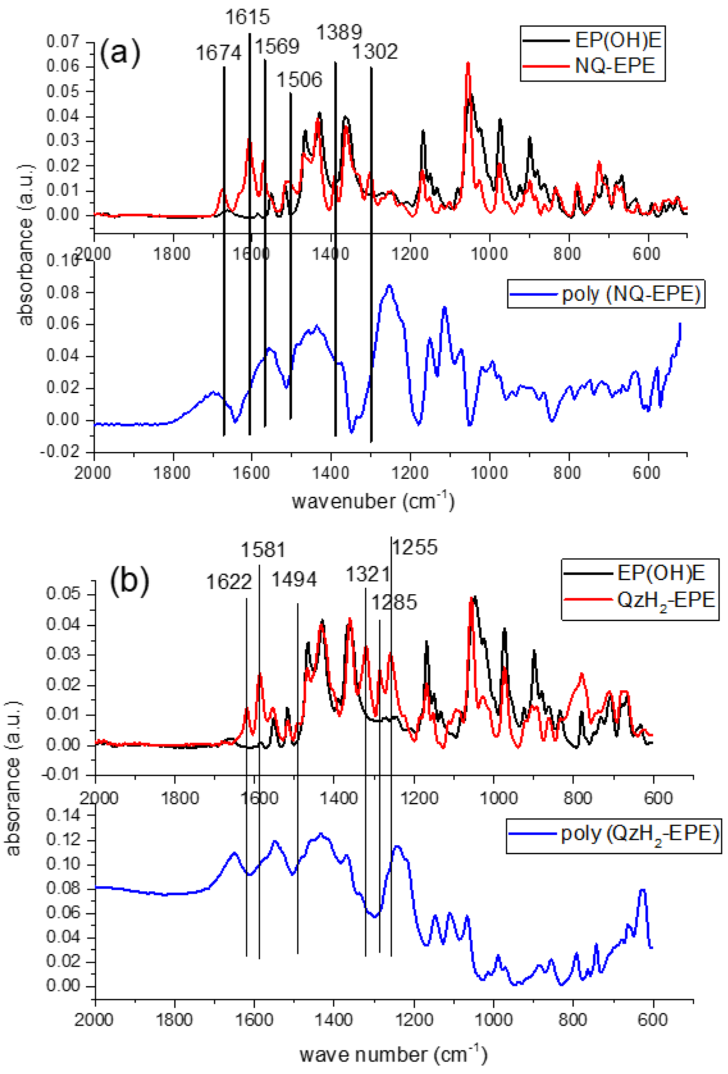

Figure S26. (a) *ex situ* ATR spectra of EP(OH)E (black), NQ-EPE trimer (red) and poly (NQ-EPE) (blue), (b) *ex situ* ATR spectra of EP(OH)E (black), QzH_2-EPE trimer (red) and poly (QzH_2-EPE) (blue). The vibrational peaks of pendants are indicated by vertical lines.

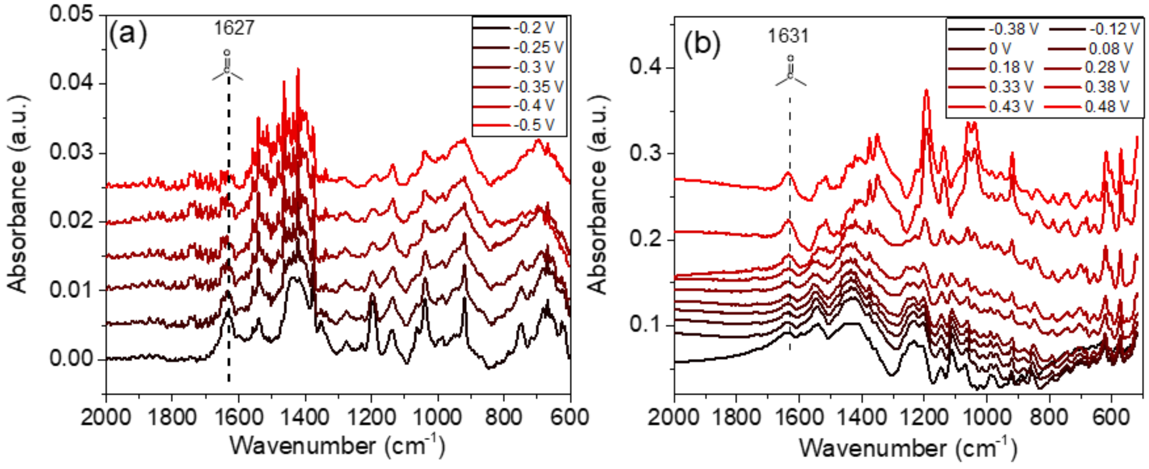

Figure S27. *In situ* FTIR spectra at different potentials of (a) poly (NQ-EPE), (b) poly (QzH_2-EPE) tested in 0.1M MeTriHTFSI/MeCN electrolyte. The characterization is conducted in a three-electrode setup.

S5: kinetic study

As the scan rate was increased the peak separation continuously increased (Figure S28-29) and at a scan rate of around 2 mV/s there was a distinct increase in the peak drift with scan rate. The peak drifts indicate that the redox reactions are too slow to keep up with the rate by which the potential is changed, *i.e.* the rates of redox conversion are of the order of 1 s^{-1}. In addition, in NQ-EPE the merged two-electron transfer process splits into two one-electron processes at high scan rates suggesting that the formation of the NQ radical intermediate, in this case, is faster than its consumption. Nevertheless, integration of the redox peaks shows that the total charge is almost constant with scan rate between 2 mV/s and 20 mV/s showing that the pendants are almost fully converted even at a scan rate of 20 mV/s (insets, Figure S28-29).

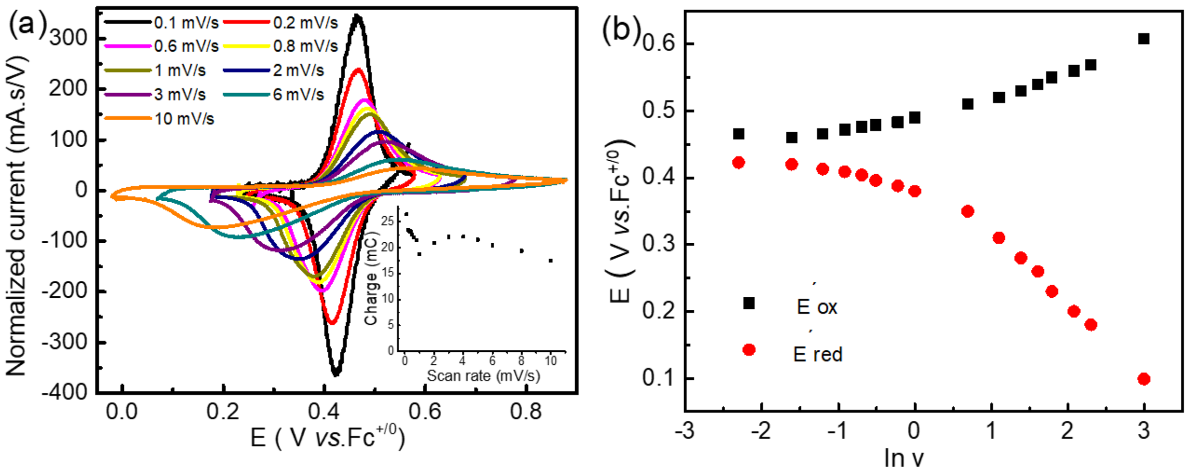

Figure S28. Kinetic study of 0.1 mg poly (QzH_2-EPE) on glassy carbon in a three-electrode setup in MeTriHTFSI. (a) Cyclic voltammograms at various scan rates, the inset figure is the integrated charge at different scan rates (b) Scan rate dependence of peak potential.

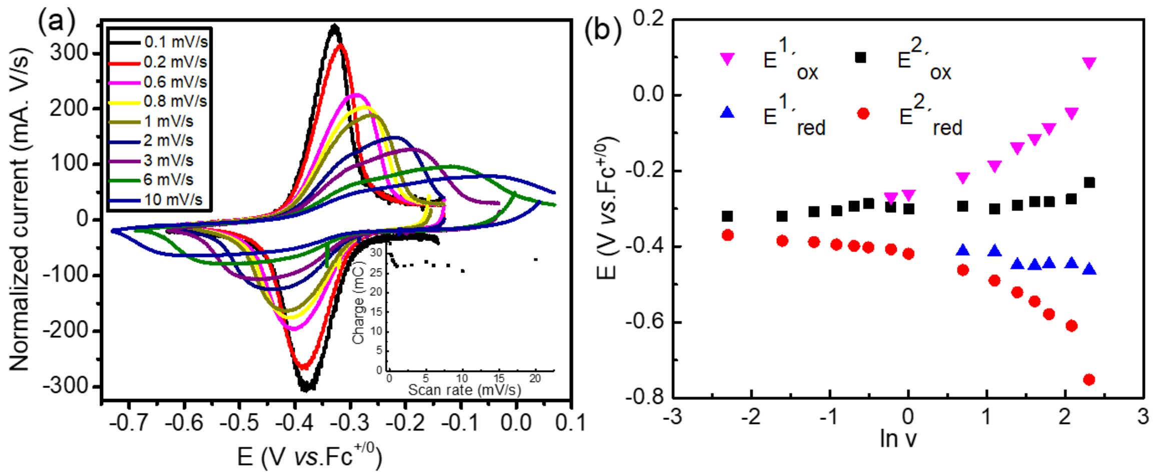

Figure S29. Kinetic study of 0.1 mg poly (NQ-EPE) on glassy carbon in a three-electrode setup in MeTriHTFSI. (a) Cyclic voltammograms at various scan rates, the inset figure is the integrated charge at different scan rates (b) Scan rate dependence of peak potential.

S5: redox match

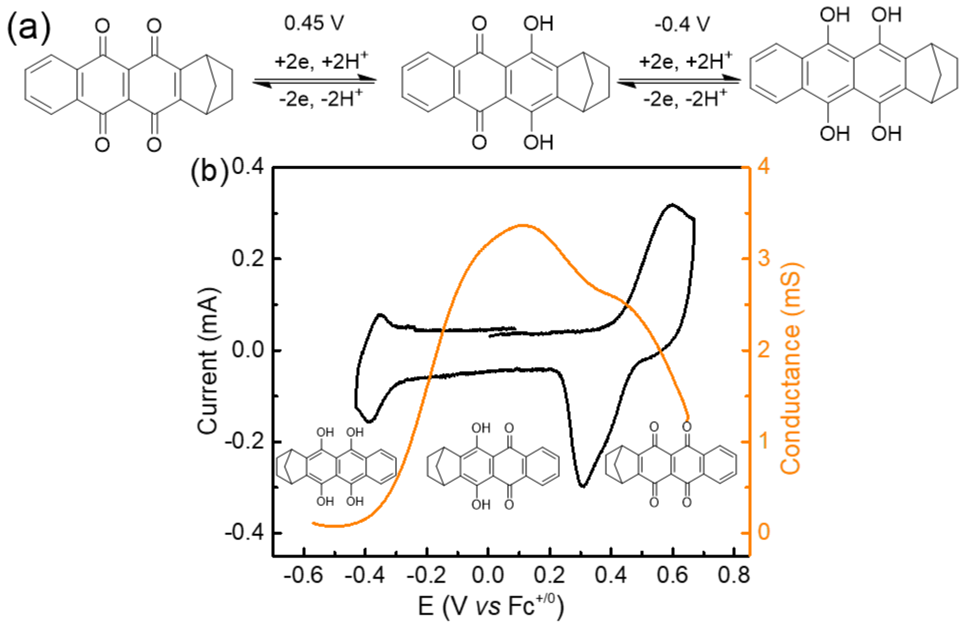

Figure S30. (a) Redox processes of Qz in MeTriHTFSI electrolyte. (b) *In situ* conductance (orange) in 0.1M MeTriHTFSI/MeCN and cyclic voltammograms (black) of poly (QzH_2-EPE) in MeTriHTFSI at a scan rate of 20 mV/s.

Section 6: Battery evaluation

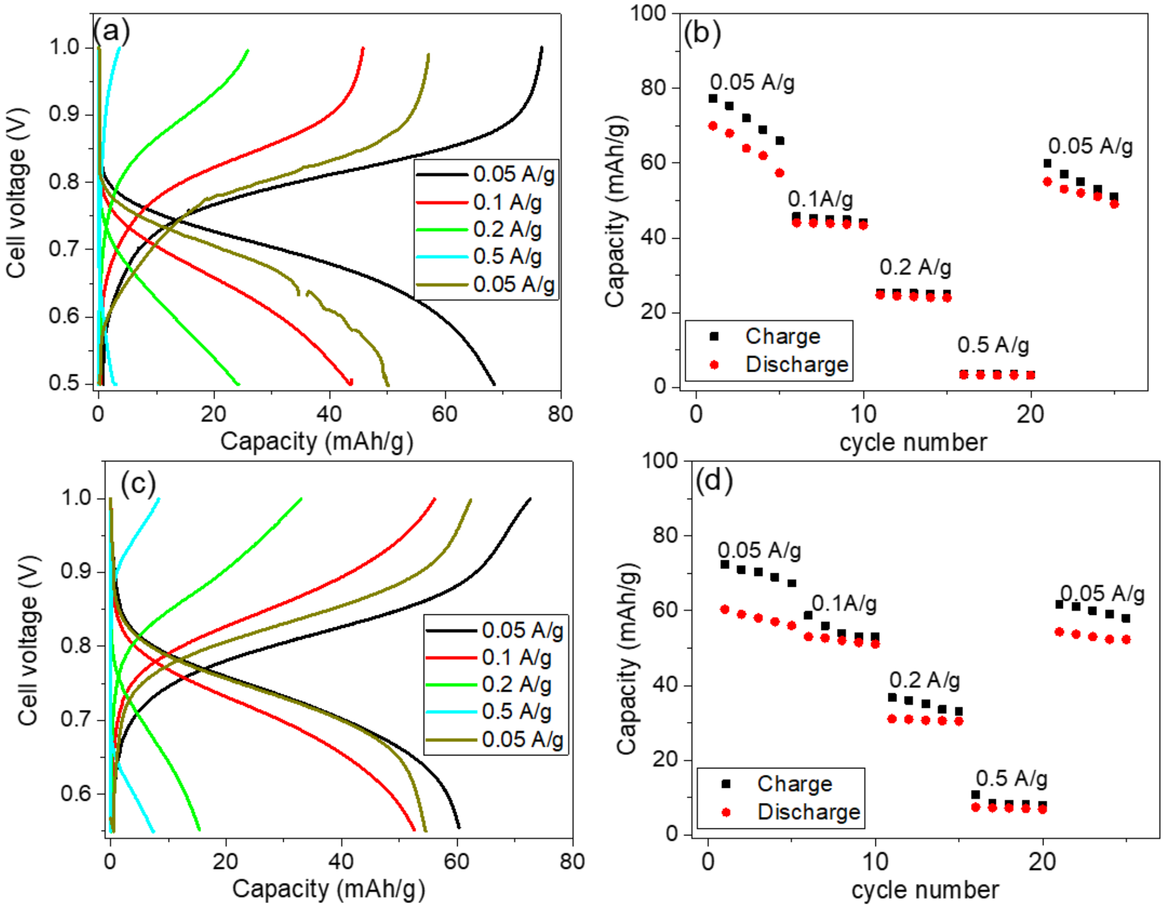

Figure S31. Galvanostatic voltage profile at different current densities and corresponding capacity of batteries using poly (NQ-EPE) as limiting material (upper panels), battery using poly (QzH_2-EPE) as limiting material (bottom panels),

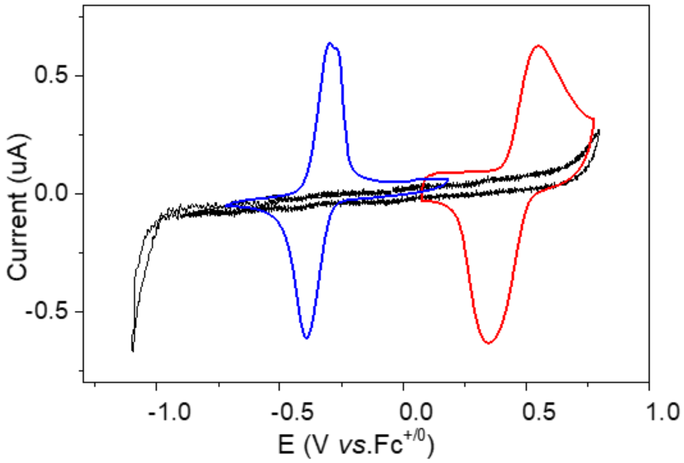

Figure S32. Cyclic voltammograms of bare glassy carbon disc (black), poly (NQ-EPE) (blue) and poly (QzH_2-EPE) (red) on glassy carbon in MeTriHTFSI electrolyte at a scan rate of 1 mV/s.

S6: *in situ* EQCM measurement

Figure S33 shows the *in situ* EQCM characterization of 10 µg poly (NQ-EPE) and 10 µg poly (QzH_2-EPE) tested in MeTriHTFSI. In the anodic scan of poly (NQ-EPE) a mass increase between -0.55 V and -0.4 V was observed, which is attributed to the backbone doping (oxidation) induced TFSI anion uptake to balance the positive charge of polymer backbone. The mass increase per molar charge in this region is 128 g/mol, which is smaller than the molar weight of TFSI (280 g/mol). It is thus concluded that the proton cation expulsion is also involved and contributes to 54% of the total mass transfer. A mass decrease between -0.4 V and -0.2 V was observed, which is attributed to the proton release from NQ/NQH_2 redox conversion. The mass decrease per molar charge in this region is -1 g/mol, which is exactly the molar mass of proton (1g/mol), suggesting that the proton repulsion is the only mass transfer process in this region. Above -0.2 V the NQ/NQH_2 redox conversion is completed, the current results from the backbone doping. However, the mass increase in this region is only 13 g/mol, which is far below the molar mass of TFSI anion. It is thus concluded that proton expulsion also occurs and contributes to

95% of the total mass transfer, becoming the dominant mass transfer process. Worthy to note that, the mass change after one cycle is only 0.6 µg, constituting 6% of the polymer mass (10 µg), indicating that NQ/NQH_2 redox conversion is stable in MeTriHTFSI. Poly (QzH_2-EPE), however, experienced a huge mass decrease (about 10 µg) during the QzH_2 oxidation peak and the polymer was totally detached from current collector due to the swelling of highly doped backbone at high doping level. To be specific, the highly charged (doped) polymer backbone requires massive TFSI anion to balance the positive charge, which can cause polymer swelling. Herein, the mass change per molar change is controversial. Worthy to note that, visible detachment only occurs on EQCM-Au current collector, not on the glassy carbon current collector. Nevertheless, we must take certain degree of swelling into consideration even on glassy carbon current collector.

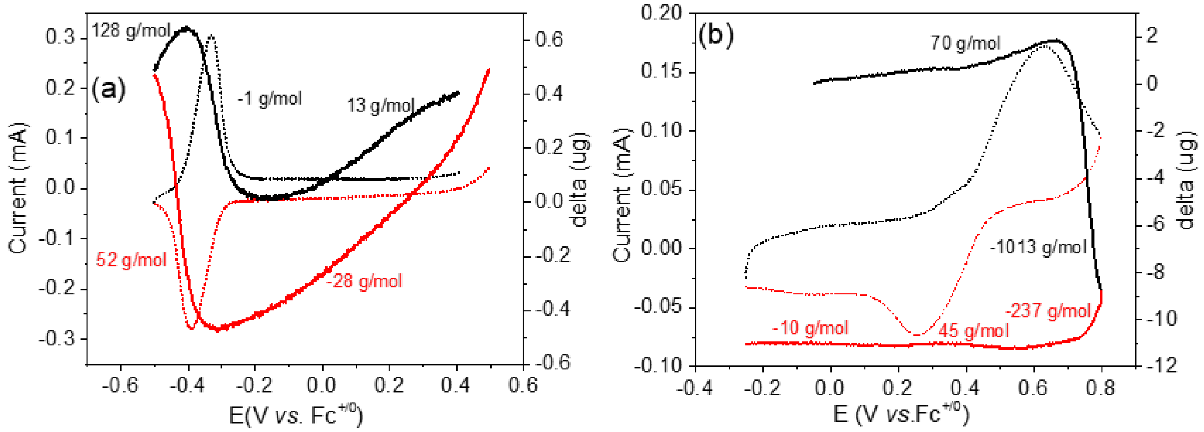

Figure S33. Cyclic voltammograms (dashed line) and corresponding mass change (solid line) of cathodic (red) and anodic (black) scan of (a) poly (NQ-EPE), (b) poly (QzH_2-EPE) on EQCM Au-electrode at a scan rate of 20 mV/s in MeTriHTFSI.

Figure S34 shows the 5 scans CVs of 10 µg poly (NQ-EPE) characterized in MeTriHTFSI. Cyclic voltammetry starts from the cathodic scan and the mass keeps increasing in the first five scans while the current is constant, suggesting that the wetting process goes on gradually in the first 5 scans.

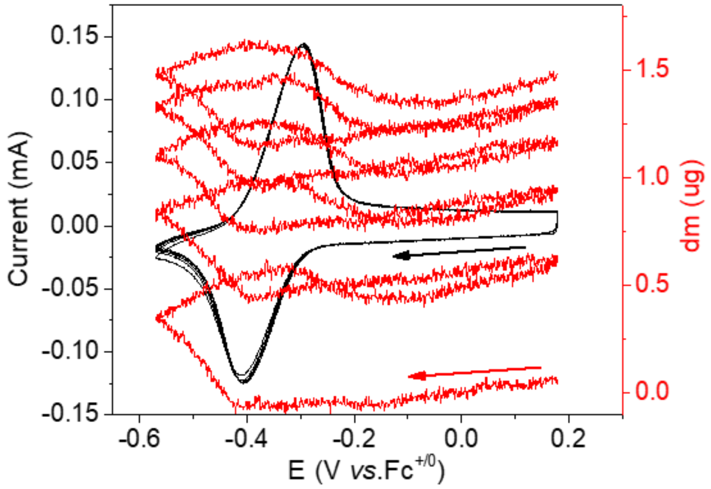

Figure S34. First five scans cyclic voltammograms (black) and corresponding mass change (red) of poly (NQ-EPE) on EQCM Au-electrode at a scan rate of 20 mV/s in MeTriHTFSI. The arrow indicates the first anodic scan.

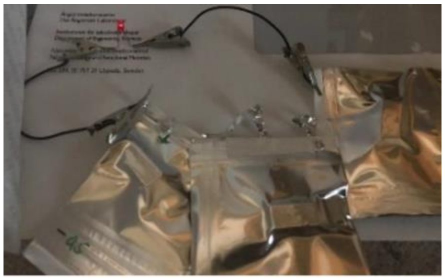

Figure S35. Photograph of a light emitting diode powdered by three batteries in series.

S6: self-discharge

The poly (QzH_2-EPE) electrode was firstly potentiostatically charged at 0.7 V for 150 s. The applied potential was then removed and open circuit potential (OCP) was allowed to relax for a certain period. After OCP relaxation the electrode was discharged at a current density 0.3 A/g.

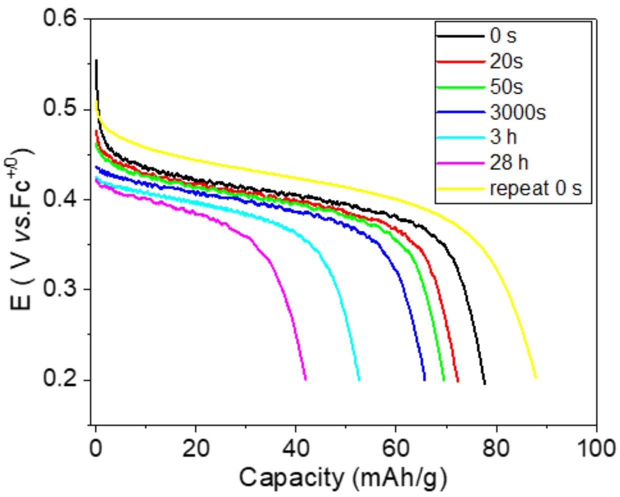

Figure S36. Self-discharge study: galvanostatic discharge curve of poly (QzH_2-EPE) on glassy carbon electrode after different relaxation time at open circuit potential state in MeTriHTFSI electrolyte.

S6: leakage current

Figure S37 shows the current of poly (QzH_2-EPE) in a three-electrode setup in response to applying a certain potential. During the first few seconds, a relatively high current is observed as a result of redox conversion or double layer charging at the electrode[7,8]. The observed high current vanished as the electrode relaxation is done and reached a new equilibrium situation. After that, a comparatively low current was observed and the current was sustained as long as the applied potential continued (Figure S37a), which is called leakage current, resulting from side reactions.

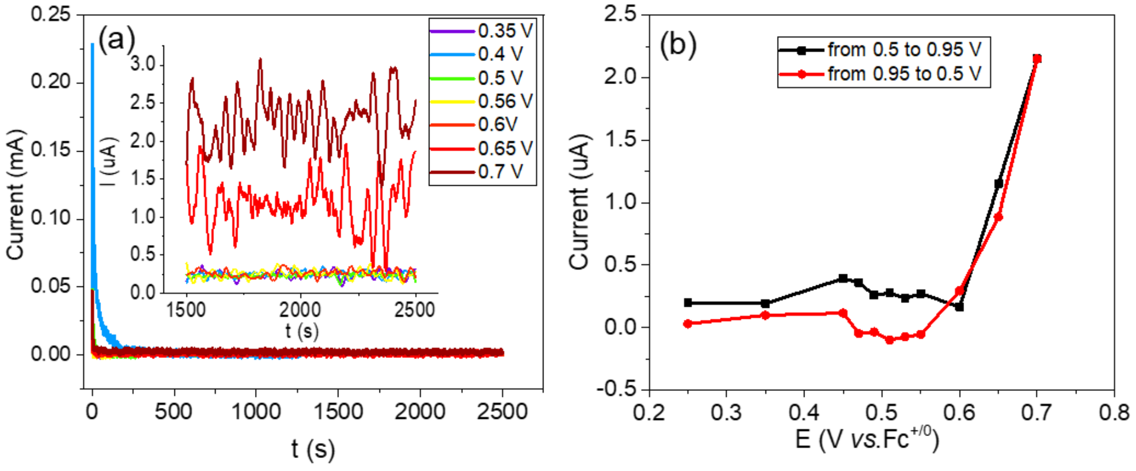

Figure S37. (a) Current response of 0.1 mg poly (QzH_2-EPE) electrode on glassy carbon in

MeTriHTFSI electrolyte when a certain potential is applied, (b) Leakage current at different potentials.

Reference

(1) Strietzel, C.; Sterby, M.; Huang, H.; Strømme, M.; Emanuelsson, R.; Sjödin, M. An Aqueous Conducting Redox-Polymer-Based Proton Battery That Can Withstand Rapid Constant-Voltage Charging and Sub-Zero Temperatures. *Angew. Chem. Int. Ed.* **2020**, *59* (24), 9631–9638. https://doi.org/10.1002/anie.202001191.

(2) Suja, T. D.; Divya, K. V. L.; Naik, L. V.; Ravi Kumar, A.; Kamal, A. Copper-Catalyzed Three-Component Synthesis of Aminonaphthoquinone–Sulfonylamidine Conjugates and in Vitro Evaluation of Their Antiproliferative Activity. *Bioorganic & Medicinal Chemistry Letters* **2016**, *26* (8), 2072–2076. https://doi.org/10.1016/j.bmcl.2016.02.071.

(3) Karlsson, C.; Strietzel, C.; Huang, H.; Sjödin, M.; Jannasch, P. Nonstoichiometric Triazolium Protic Ionic Liquids for All-Organic Batteries. *ACS Appl. Energy Mater.* **2018**, *1* (11), 6451–6462. https://doi.org/10.1021/acsaem.8b01389.

(4) Karlsson, C.; Huang, H.; Strømme, M.; Gogoll, A.; Sjödin, M. Ion- and Electron Transport in Pyrrole/Quinone Conducting Redox Polymers Investigated by In Situ Conductivity Methods. *Electrochimica Acta* **2015**, *179*, 336–342. https://doi.org/10.1016/j.electacta.2015.02.193.

(5) Sterby, M.; Emanuelsson, R.; Huang, X.; Gogoll, A.; Strømme, M.; Sjödin, M. Characterization of PEDOT-Quinone Conducting Redox Polymers for Water Based Secondary Batteries. *Electrochimica Acta* **2017**, *235*, 356–364. https://doi.org/10.1016/j.electacta.2017.03.068.

(6) Karlsson, C.; Huang, H.; Strømme, M.; Gogoll, A.; Sjödin, M. Ion- and Electron Transport in Pyrrole/Quinone Conducting Redox Polymers Investigated by In Situ Conductivity Methods. *Electrochimica Acta* **2015**, *179*, 336–342. https://doi.org/10.1016/j.electacta.2015.02.193.

(7) Vadivel, N. R.; Ha, S.; He, M.; Dees, D.; Trask, S.; Polzin, B.; Gallagher, K. G. On Leakage Current Measured at High Cell Voltages in Lithium-Ion Batteries. *J. Electrochem. Soc.* **2017**, *164* (2), A508–A517. https://doi.org/10.1149/2.1341702jes.

(8) Wessells, C.; Ruffo, R.; Huggins, R. A.; Cui, Y. Investigations of the Electrochemical Stability of Aqueous Electrolytes for Lithium Battery Applications. *Electrochem. Solid-State Lett.* **2010**, *13* (5), A59. https://doi.org/10.1149/1.3329652.
